# Association Between Acoustic Features and Neuropsychological Test Performance in the Framingham Heart Study: Observational Study

**DOI:** 10.2196/42886

**Published:** 2022-12-22

**Authors:** Huitong Ding, Amiya Mandapati, Cody Karjadi, Ting Fang Alvin Ang, Sophia Lu, Xiao Miao, James Glass, Rhoda Au, Honghuang Lin

**Affiliations:** 1 Department of Anatomy and Neurobiology Chobanian & Avedisian School of Medicine Boston University Boston, MA United States; 2 Department of Religious Studies Brown University Providence, RI United States; 3 The Warren Alpert Medical School Brown University Providence, RI United States; 4 The Framingham Heart Study Chobanian & Avedisian School of Medicine Boston University Framingham, MA United States; 5 Slone Epidemiology Center Chobanian & Avedisian School of Medicine Boston University Boston, MA United States; 6 Department of Epidemiology School of Public Health Boston University Boston, MA United States; 7 Innovation Research Institute of Traditional Chinese Medicine Shanghai University of Traditional Chinese Medicine Shanghai China; 8 Computer Science and Artificial Intelligence Laboratory Massachusetts Institute of Technology Cambridge, MA United States; 9 Department of Neurology Chobanian & Avedisian School of Medicine Boston University Boston, MA United States; 10 Department of Medicine University of Massachusetts Chan Medical School Worcester, MA United States

**Keywords:** mild cognitive impairment, digital voice, neuropsychological test, association, prediction

## Abstract

**Background:**

Human voice has increasingly been recognized as an effective indicator for the detection of cognitive disorders. However, the association of acoustic features with specific cognitive functions and mild cognitive impairment (MCI) has yet to be evaluated in a large community-based population.

**Objective:**

This study aimed to investigate the association between acoustic features and neuropsychological (NP) tests across multiple cognitive domains and evaluate the added predictive power of acoustic composite scores for the classification of MCI.

**Methods:**

This study included participants without dementia from the Framingham Heart Study, a large community-based cohort with longitudinal surveillance for incident dementia. For each participant, 65 low-level acoustic descriptors were derived from voice recordings of NP test administration. The associations between individual acoustic descriptors and 18 NP tests were assessed with linear mixed-effect models adjusted for age, sex, and education. Acoustic composite scores were then built by combining acoustic features significantly associated with NP tests. The added prediction power of acoustic composite scores for prevalent and incident MCI was also evaluated.

**Results:**

The study included 7874 voice recordings from 4950 participants (age: mean 62, SD 14 years; 4336/7874, 55.07% women), of whom 453 were diagnosed with MCI. In all, 8 NP tests were associated with more than 15 acoustic features after adjusting for multiple testing. Additionally, 4 of the acoustic composite scores were significantly associated with prevalent MCI and 7 were associated with incident MCI. The acoustic composite scores can increase the area under the curve of the baseline model for MCI prediction from 0.712 to 0.755.

**Conclusions:**

Multiple acoustic features are significantly associated with NP test performance and MCI, which can potentially be used as digital biomarkers for early cognitive impairment monitoring.

## Introduction

Alzheimer disease (AD) is a chronic neurodegenerative disease characterized behaviorally by memory loss, language impairment, motor problems, loss of executive function, and emotional distress, which can progress to severe levels. There are currently no definitive disease-modifying treatment methods [1], but general consensus is that early detection is critical. Interventions through the reduction of modifiable risk factors may serve to delay, attenuate, or even prevent disease onset and progression [2,3]. Mild cognitive impairment (MCI) is a prodromal stage of AD in which cognitive decline does not affect essential functions of daily life [4], but some individuals may have difficulty remembering events and situations, as well as problems with executive function [5]. The detection of MCI is critical to initiate current interventions that may slow down the neurodegenerative process [6] and participate in clinical trials that may lead to effective treatments.

At present, diagnosis relies largely on some combination of clinical examination [7], neuroimaging (eg, magnetic resonance imaging [8] and positron emission tomography [9]), and neuropsychological (NP) testing [10]. Fluid biomarkers are being developed as alternatives to expensive and burdensome imaging through the analysis of cerebrospinal fluid [11] and blood analysis [12]. Although substantial advancements have been made in developing pathological indicators of AD (eg, imaging and fluid biomarkers), surprisingly little has been done to develop better cognitive assessment methods beyond the traditional NP tests. The well-documented heterogeneity of cognition has made the accurate diagnosis of MCI elusive [13,14].

Producing speech is a cognitively complex task [15], and recording speech is relatively easy given the widespread accessibility to recording devices. Research has found that language deficits may occur in the prodromal stages [16] of cognitive impairment, which present years prior to clinical diagnosis [17,18], potentially making it an effective indicator for MCI. Meanwhile, the development of speech feature extraction technology offers the possibility of quantifying voice signal properties from multiple dimensions. It empowers the comprehensive description of specific pathologies by voice features. The lexical, acoustic, and syntactic features extracted from the human voice have been shown to be significantly associated with dementia [19,20]. Using voice-based biomarkers as a screening method presupposes an economic solution for the early diagnosis of MCI. Increasing evidence suggests that the human voice could be used as a powerful resource to derive pathologically appropriate biomarkers for dementia. Multiple acoustic biomarkers have also been related to the future risk of dementia [21].

Applying the findings of earlier research to a general population, however, is difficult due to the small sample sizes and use of cognitive assessment protocols that are not sufficiently comprehensive. Further, voice analyses that include linguistic features are difficult to generalize to other languages. There remains a paucity of research determining the relationship between acoustic features and NP tests that span across multiple cognitive domains. In addition, a comprehensive characterization of acoustic features that are associated with incident MCI is warranted. The objective of this study was to investigate the association of acoustic features and different NP test scores across cognitive domains and how they compare in identifying prevalent and incident MCI in the Framingham Heart Study (FHS) community-based cohort.

## Methods

### Sample Selection

The original sample included 9253 observations from 5189 participants who completed at least one NP assessment that was voice recorded. A subset of participants had multiple recordings over the course of the study period. Each digital voice recording and the corresponding NP tests were treated as 1 observation. Exclusion criteria included those observations with missing education information (n=492), prevalent dementia (n=313), flagged as potential MCI but have not gone through dementia review (n=551), and those whose voice recording was less than 10 minutes in length (n=23).

### Ethics Approval

The Institutional Review Board of the Boston University Medical Campus approved the procedures and protocols of the Framingham Heart Study (FHS is H-32132). All participants provided written informed consent.

### NP Assessment

The details of FHS NP test administration have been reported previously [22]. Multiple cognitive domains are measured by 18 different tests [23-27] including verbal memory, verbal fluency, visual memory, attention and concentration, executive function, abstract reasoning, visuoperceptual organization, and language, as is illustrated in Table 1.

**Table 1:** Cognitive domain and corresponding neuropsychological (NP) tests.

Cognitive domain	NP test
Verbal memory	Logical Memory—Immediate Recall Logical Memory—Delayed Recall Logical Memory—Recognition Paired Associate Learning—Immediate Recall Paired Associate Learning—Delayed Recall Paired Associate Learning—Recognition
Visual memory	Visual Reproduction—Immediate Recall Visual Reproduction—Delayed Recall Visual Reproduction—Recognition
Attention and concentration	Digit Span—Forward Trail Making Test A
Executive function	Digit Span—Backward Trail Making Test B
Abstract reasoning	Similarities
Language	Boston Naming Test—30-item version
Visuoperceptual organization	Hooper Visual Organization Test
Verbal fluency	Controlled Oral Word Association Test Category Naming Test—Animal

### Voice Recordings

Since 2005, the FHS has been digitally recording all spoken responses during NP test administration, which encompasses the verbal interactions between the tester and the participant. This study included digital voice recordings obtained from September 2005 to March 2020. OpenSMILE software (version 2.1.3) [28] was used to extract an acoustic feature set [29], which contains 65 low-level descriptors (LLDs) from these recordings. This acoustic feature set covers a broad range of information of the voice recordings including pitch, voice quality, loudness, signal energy, waveform, auditory, fast Fourier transform spectrum, spectral, and cepstral, which has been described in detail in a prior study [30]. The feature set has also been used in many fields, such as speech processing, music information retrieval, and emotion recognition [31]. The description of these features is summarized in Table S1 in Multimedia Appendix 1. More details of these features can be found in the previous publication [30]. There are some audio recordings with 1 channel (mono; n=4738), and the others were recorded with 2 channels (stereo; n=3136). For the recordings with 2 channels, we included the first channel in the analysis. Each recording was divided into segments of 20 milliseconds using a sliding window approach with a shifting size of 10 milliseconds. The LLD features were extracted from these segments. For each recording, we further computed the mean of each LLD feature to capture its high-level statistical features, which were then normalized.

### Ascertainment of MCI

The cognitive status of FHS participants included assessments by NP tests. For those identified with possible cognitive impairment, NP tests were administered on average about every 1 to 2 years. When potential cognitive impairment decline was present, a clinical review was conducted by a panel with at least one neurologist and one neuropsychologist. MCI diagnosis was determined by the review panel, which required that the participant exhibit evidence of a decline in cognitive performance in 1 or more cognitive domains, have no records indicating functional decline, and do not meet the criteria for dementia [32]. The Clinical Dementia Rating scale [33] was used to quantify the severity of impairment. In all, 2 outcomes were considered in this study. The prevalent MCI cases were subjects who were diagnosed with MCI before or at the time when the voice was recorded. The incident MCI cases were all subjects who were cognitively intact at baseline but were diagnosed with MCI during the follow-up.

### Statistical Analyses

To compare the difference between demographics and standard NP test scores in MCI and normal control groups, Wilcoxon rank sum test was used for continuous variables [34]. The chi-square test was used to compare differences in frequencies for categorical variables [35]. Log transformations were applied for NP tests with skewed distributions to normalize them. Normalized values of NP tests and acoustic features were used in the analysis. Linear mixed-effects models were used to quantify the association between each acoustic feature and NP tests [36].

A set of acoustic composite scores was generated by regressing each NP test against the group of acoustic features that were significantly associated with each NP test. The acoustic composite score is a weighted combination of acoustic features. The weight of each acoustic feature in the composite score was derived by training a linear mixed-effects effect model. For participant *i*, the acoustic composite score of an NP test is defined as



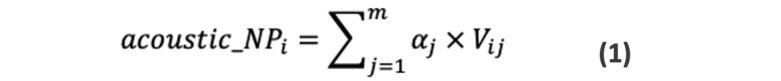



where *m* is the number of acoustic features significantly associated with the NP test, α*_j_* is the estimate of effect size for the acoustics feature *j* derived from the linear mixed-effects effect model, and *V_ij_* is the normalized acoustics feature *j* for participant *i*. The association between normalized acoustic composite scores with corresponding NP tests was assessed by linear mixed-effects models.

The association of normalized acoustic composite scores with prevalent MCI was assessed by logistic regression models. Based on the regression coefficients, the odds ratios (ORs) and 95% CIs were estimated.

To determine the relationship between acoustic composite scores and incident MCI, participants whose age at the voice recording was <60 years (n=2718) and those with prevalent MCI (n=222) were excluded. The first observation of each participant was included in this analysis. The association between acoustic composite scores with incident MCI was quantified by Cox proportional hazards models (censored at the last date of contact or death) [37]. All models were adjusted for age, sex, and education. Bonferroni correction was used to adjust for multiple tests.

We further evaluated the added predictability of the acoustic composite score for incident MCI. Receiver operating characteristic (ROC) analysis was performed to estimate the area under the curve (AUC) using a random forest model. A baseline model was constructed using age, sex, and education as predictors. A second model was constructed using these predictors and additional acoustic composite scores that were found to be significantly related to specific NP tests. The mean AUC of 10-fold cross-validation was computed for each model for comparison. We also performed a secondary analysis by including NP tests and clinical risk factors in the prediction of incident MCI. The statistical analyses were performed using Python software (version 3.9.7; Python Software Foundation).

## Results

Our study included 7874 observations from 4950 participants of FHS (age: mean 62, SD 14 years; 4336/7874, 55.07% women; 4279/7874, 54.34% self-reported college-level education or higher). Most participants (2657/4950, 53.68%) had 1 voice recording. Some participants (1775/4950, 35.86%) had 2 recordings, and the remaining participants (518/4950, 10.46%) had 3 or more recordings. Among these observations, 453 of these observations were diagnosed with MCI. The details of sample characteristics are shown in Table 2.

We examined the association of acoustic features with NP tests. As shown in Table 3, eight NP tests (Visual Reproduction—Immediate Recall [VRi], Visual Reproduction—Delayed Recall [VRd], Digit Span—Forward, Digit Span—Backward, Similarities [SIM], Boston Naming Test—30-item version, Controlled Oral Word Association Test [FAS], and Category Naming Test—Animal) were associated with more than 15 acoustic features. The *mfcc_sma* [2] was the most significant acoustic feature with 3 NP tests (Boston Naming Test—30-item version, FAS, and Category Naming Test—Animal) after Bonferroni correction (*P*<7.7 × 10^–4^) that represents Mel-frequency cepstral coefficient (MFCC) 2. The details of associations between acoustic features and NP tests are fully depicted in Table S2 in Multimedia Appendix 1. We also summarized the acoustic features that were significantly associated with NP tests across cognitive domains in Table S3 in Multimedia Appendix 1. It shows that visual memory was associated with 49 acoustic features. Each cognitive domain had an average of 28 associated acoustic features. In the sensitivity analysis, besides age, sex, and education, we further included employment as an additional covariate to examine the stability of the association between acoustic features and NP tests. As shown in Table S4 in Multimedia Appendix 1, similar acoustic features were found to be associated with NP tests. In addition, we also examined the correlation between acoustic features and NP tests collected at the same time and a later time. For each NP test conducted at the first exam, we compared its correlation with acoustic features collected at the first exam and the second exam. As shown in Table S5 in Multimedia Appendix 1, only moderate changes were observed between the 2 exams.

Acoustic composite scores were also generated using the significant acoustic features for each NP test. As shown in Table 4, all these scores were significantly associated with their corresponding NP tests.

We then performed association analysis of acoustic composite scores with prevalent MCI. Table 5 shows that 4 acoustic composite scores (*acoustic_LMr*, *acoustic_TrailsB*, *acoustic_FAS*, and *acoustic_CNT_Animal*) were significantly associated with prevalent MCI (OR ranging from 0.69 to 1.23; *P*<3.1 × 10^–3^). Lower acoustics composite scores (*acoustic_TrailsB*, *acoustic_FAS*, and *acoustic_CNT_Animal*) were associated with higher OR of MCI after adjusting for age, sex, and education (*P*<3.1 × 10^–3^). The most significant acoustic composite score was for FAS Animal test (*P*=2.3 × 10^–7^).

We further examined the association of acoustic composite scores with incident MCI by restricting the analysis to 2010 participants who were aged ≥60 years. Among them, 145 participants have incident MCI. As shown in Table 6, the acoustic composite scores for Logical Memory—Immediate Recall (LMi), VRi, VRd, Visual Reproduction—Recognition (VRr), SIM, Trail Making Test B (TrailsB), and Hooper Visual Organization Test (HVOT) tests were significantly associated with incident MCI (*P*<3.1 × 10^–3^). Higher acoustic composite scores for VRi, VRd, SIM, and TrailsB tests were associated with higher MCI risk. The other 3 scores were negatively associated with MCI risk with hazard ratio lower than 1 after adjusting for age, sex, and education. We further built 2 Cox regression models for incident MCI to show the contribution of acoustic features. Model 1 includes age, sex, and education as predictors. Model 2 includes age, sex, education, and all significant associated acoustic composite scores with incident MCI. The change in Akaike information criterion [38] with the addition of acoustic composite scores to the model was calculated. We observed a smaller Akaike information criterion for model 2, suggesting that the model better fit the prediction.

**Figure 1:**
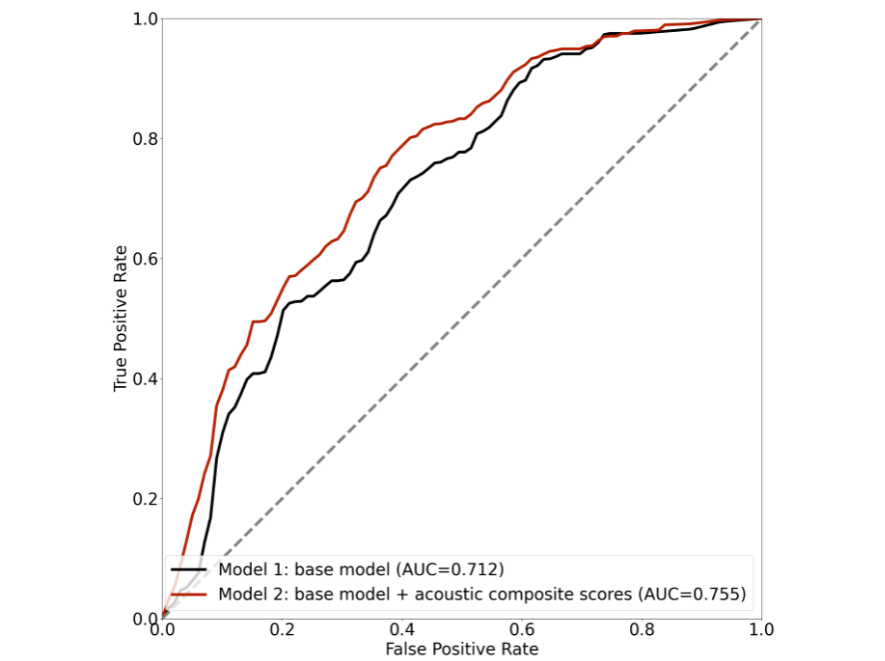
The receiver operating characteristic curves of 2 models to predict incident mild cognitive impairment. AUC: area under the curve.

The added predictive power of *acoustic_LMi*, *acoustic_VRi*, *acoustic_VRd*, *acoustic_VRr*, *acoustic_SIM*, *acoustic_TrailsB*, and *acoustic_HVOT* for incident MCI were evaluated by comparing the AUC of different models. Model 1 only included age, sex, and education as the predictors of incident MCI. Besides age, sex, and education, Model 2 included 7 composite scores that were significantly associated with incident MCI as the predictors. Model 3 included age, sex, education, and 18 NP tests as predictors. Figure 1 shows that the AUC of MCI prediction can be improved from 0.712 (model 1) to 0.755 (model 2) by including acoustic composite scores of LMi, VRi, VRd, VRr, SIM, TrailsB, and HVOT tests. As shown in Figure S1 in Multimedia Appendix 1, the model with NP tests reached AUC=0.761, which is comparable to the one including demographic factors and acoustic composite scores (DeLong test *P*=.97). However, both models showed significant improvement over model 1 that included only demographic factors (DeLong test *P*=.03 and *P*=.03 for model 2 and model 3, respectively). These results indicate that the acoustics composite scores have similar predictive power to traditional NP tests. Compared to the burden of conducting NP tests, the prediction model based on acoustic features relied minimally on NP expertise; these results suggest the feasibility of developing real-time cognitive screening tools.

**Table 2:** Baseline characteristics.

Variable		Total observation (N=7874)	MCI^a^ (n=453)	NC^b^ (n=7421)	*P* value^c^	
Age (years), mean (SD)		62 (14)	81 (8)	61 (14)	<.001	
**Gender, n (%)**						.84
	Women	4336 (55.07)	252 (55.63)	4084 (55.03)		
	Men	3538 (44.93)	201 (44.37)	3337 (44.97)		
**Education, n (%)**						<.001
	No high school	202 (2.57)	53 (11.70)	149 (2.01)		
	High school	1443 (18.33)	118 (26.05)	1295 (17.45)		
	Some college	1950 (24.77)	134 (29.58)	1816 (24.47)		
	College and higher	4279 (54.34)	148 (32.67)	4161 (56.07)		
**NP^d^ test score, mean (SD)**						
	LMi^e^	12.35 (3.62)	8.53 (3.76)	12.58 (3.48)	<.001	
	LMd^f^	11.36 (3.83)	6.93 (4.11)	11.62 (3.65)	<.001	
	LMr^g^	9.52 (1.28)	8.59 (1.72)	9.57 (1.23)	<.001	
	VRi^h^	8.61 (2.91)	4.48 (2.23)	8.85 (2.76)	<.001	
	VRd^i^	7.91 (3.17)	3.11 (2.30)	8.19 (2.99)	<.001	
	VRr^j^	3.11 (1.01)	1.89 (1.06)	3.18 (0.96)	<.001	
	PASi^k^	14.45 (3.58)	10.02 (2.79)	14.71 (3.45)	<.001	
	PASd^l^	8.56 (1.47)	6.56 (1.60)	8.68 (1.38)	<.001	
	PASr^m^	9.82 (0.64)	8.83 (1.74)	9.88 (0.45)	<.001	
	DSf^n^	6.71 (1.31)	6.06 (1.20)	6.75 (1.30)	<.001	
	DSb^o^	4.92 (1.30)	4.12 (1.01)	4.97 (1.30)	<.001	
	SIM^p^	16.83 (3.61)	12.63 (4.30)	17.08 (3.40)	<.001	
	BNT30^q^	27.22 (2.81)	23.66 (4.14)	27.43 (2.56)	<.001	
	TrailsA^r^	0.42 (0.15)	0.66 (0.21)	0.40 (0.14)	<.001	
	TrailsB^s^	0.85 (0.34)	1.54 (0.50)	0.82 (0.29)	<.001	
	HVOT^t^	3.26 (0.15)	3.06 (0.22)	3.27 (0.13)	<.001	
	FAS^u^	39.85 (12.52)	28.76 (11.68)	40.50 (12.26)	<.001	
	CNT_Animal^v^	19.48 (5.68)	12.22 (4.37)	19.91 (5.46)	<.001	

^a^MCI: mild cognitive impairment.

^b^NC: normal control.

^c^Significant associations were claimed if *P*<.05/18≈.002.

^d^NP: neuropsychological.

^e^LMi: Logical Memory—Immediate Recall.

^f^LMd: Logical Memory—Delayed Recall.

^g^LMr: Logical Memory—Recognition.

^h^VRi: Visual Reproduction—Immediate Recall.

^i^VRd: Visual Reproduction—Delayed Recall.

^j^VRr: Visual Reproduction—Recognition.

^k^PASi: Paired Associate Learning—Immediate Recall.

^l^PASd: Paired Associate Learning—Delayed Recall.

^m^PASr: Paired Associate Learning—Recognition.

^n^DSf: Digit Span—Forward.

^o^DSb: Digit Span—Backward.

^p^SIM: Similarities.

^q^BNT30: Boston Naming Test—30-item version.

^r^TrailsA: Trail Making Test A.

^s^TrailsB: Trail Making Test B.

^t^HVOT: Hooper Visual Organization Test.

^u^FAS: Controlled Oral Word Association Test.

^v^CNT_Animal: Category Naming Test—Animal.

**Table 3:** The most significant acoustic feature for each neuropsychological (NP) test.

NP test	Significant acoustic features, n	The most significant acoustic feature	Effect size	SE	*P* value^a^
LMi^b^	7	audSpec_Rfilt_sma [25]	0.0490	0.0095	2.7 × 10^–^^7^
LMd^c^	3	audSpec_Rfilt_sma [25]	0.0402	0.0094	1.9 × 10^–^^5^
LMr^d^	3	audSpec_Rfilt_sma [23]	0.0397	0.0108	2.3 × 10^–^^4^
VRi^e^	49	mfcc_sma [11]	0.1409	0.0082	8.4 × 10^–^^66^
VRd^f^	43	mfcc_sma [11]	0.1137	0.0082	3.7 × 10^–^^44^
VRr^g^	10	pcm_fftMag_spectralRollOff75.0_sma	–0.0358	0.0095	1.7 × 10^–^^4^
PASi^h^	0	N/A^i^	N/A	N/A	N/A
PASd^j^	0	N/A	N/A	N/A	N/A
PASr^k^	7	audSpec_Rfilt_sma [1]	–0.0709	0.0112	2.3 × 10^–^^10^
DSf^l^	44	audSpec_Rfilt_sma [6]	0.0898	0.0107	4.8 × 10^–^^17^
DSb^m^	30	audSpec_Rfilt_sma [5]	0.0624	0.0110	1.2 × 10^–^^8^
SIM^n^	24	pcm_fftMag_spectralRollOff75.0_sma	–0.0530	0.0084	2.4 × 10^–^^10^
BNT30^o^	23	mfcc_sma [2]	0.0433	0.0069	3.2 × 10^–^^10^
TrailsA^p^	15	pcm_fftMag_spectralSkewness_sma	–0.0363	0.0075	1.4 × 10^–^^6^
TrailsB^q^	1	pcm_fftMag_spectralSkewness_sma	–0.0269	0.0074	3.1 × 10^–^^4^
HVOT^r^	5	F0final_sma	–0.0472	0.0093	3.6 × 10^–^^7^
FAS^s^	26	mfcc_sma [2]	0.0534	0.0073	3.6 × 10^–^^13^
CNT_Animal^t^	34	mfcc_sma [2]	0.0715	0.0082	2.6 × 10^–^^18^

^a^Significant associations were claimed if *P*<.05/65≈7.7 × 10^–4^.

^b^LMi: Logical Memory—Immediate Recall.

^c^LMd: Logical Memory—Delayed Recall.

^d^LMr: Logical Memory—Recognition.

^e^VRi: Visual Reproduction—Immediate Recall.

^f^VRd: Visual Reproduction—Delayed Recall.

^g^VRr: Visual Reproduction—Recognition.

^h^PASi: Paired Associate Learning—Immediate Recall.

^i^N/A: not applicable.

^j^PASd: Paired Associate Learning—Delayed Recall.

^k^PASr: Paired Associate Learning—Recognition.

^l^DSf: Digit Span—Forward.

^m^DSb: Digit Span—Backward.

^n^SIM: Similarities.

^o^BNT30: Boston Naming Test—30-item version.

^p^TrailsA: Trail Making Test A.

^q^TrailsB: Trail Making Test B.

^r^HVOT: Hooper Visual Organization Test.

^s^FAS: Controlled Oral Word Association Test.

^t^CNT_Animal: Category Naming Test—Animal.

**Table 4:** Association between acoustic composite scores and corresponding neuropsychological tests.

Acoustic composite score	Effect size	SE	*P* value^a^
acoustic_LMi^b^	0.0579	0.0094	6.6 × 10^–^^10^
acoustic_LMd^c^	0.0310	0.0095	1.1 × 10^–^^3^
acoustic_LMr^d^	0.0358	0.0105	6.8 × 10^–^^4^
acoustic_VRi^e^	0.1510	0.0086	3.3 × 10^–^^69^
acoustic_VRd^f^	0.1079	0.0086	6.5 × 10^–^^36^
acoustic_VRr^g^	–0.0291	0.0098	3.0 × 10^–^^3^
acoustic_PASr^h^	0.0841	0.0114	1.3 × 10^–^^13^
acoustic_DSf^i^	0.1298	0.0097	1.8 × 10^–^^40^
acoustic_DSb^j^	0.0553	0.0102	6.2 × 10^–^^8^
acoustic_SIM^k^	0.0719	0.0089	5.1 × 10^–^^16^
acoustic_BNT30^l^	0.0458	0.0071	1.4 × 10^–^^10^
acoustic_TrailsA^m^	0.0408	0.0088	3.0 × 10^–^^6^
acoustic_TrailsB^n^	–0.0269	0.0075	3.1 × 10^–^^4^
acoustic_HVOT^o^	0.0284	0.0090	1.7 × 10^–^^3^
acoustic_FAS^p^	0.0827	0.0079	1.4 × 10^–^^25^
acoustic_CNT_Animal^q^	0.0529	0.0098	6.5 × 10^–^^8^

^a^Significant associations were claimed if *P*<.05/16≈3.1 × 10^–3^.

^b^LMi: Logical Memory—Immediate Recall.

^c^LMd: Logical Memory—Delayed Recall.

^d^LMr: Logical Memory—Recognition.

^e^VRi: Visual Reproduction—Immediate Recall.

^f^VRd: Visual Reproduction—Delayed Recall.

^g^VRr: Visual Reproduction—Recognition.

^h^PASr: Paired Associate Learning—Recognition.

^i^DSf: Digit Span—Forward.

^j^DSb: Digit Span—Backward.

^k^SIM: Similarities.

^l^BNT30: Boston Naming Test—30-item version.

^m^TrailsA: Trail Making Test A.

^n^TrailsB: Trail Making Test B.

^o^HVOT: Hooper Visual Organization Test.

^p^FAS: Controlled Oral Word Association Test.

^q^CNT_Animal: Category Naming Test—Animal.

**Table 5:** Association between acoustic composite scores and prevalent mild cognitive impairment.

Acoustic composite score	Odds ratio (95% CI)	*P* value^a^
acoustic_LMi^b^	1.09 (0.94-1.26)	2.6 × 10^–1^
acoustic_LMd^c^	1.14 (0.99-1.31)	7.4 × 10^–2^
acoustic_LMr^d^	1.23 (1.08-1.40)	*1.6 × 10* ^–3^
acoustic_VRi^e^	1.05 (0.92-1.19)	4.7 × 10^–1^
acoustic_VRd^f^	1.07 (0.94-1.21)	3.2 × 10^–1^
acoustic_VRr^g^	0.94 (0.80-1.10)	4.6 × 10^–1^
acoustic_PASr^h^	0.9 (0.81-0.99)	3.6 × 10^–2^
acoustic_DSf^i^	1.17 (1.04-1.32)	1.1 × 10^–2^
acoustic_DSb^j^	0.94 (0.83-1.07)	3.5 × 10^–1^
acoustic_SIM^k^	0.94 (0.84-1.06)	3.1 × 10^–1^
acoustic_BNT30^l^	0.92 (0.82-1.04)	2.0 × 10^–1^
acoustic_TrailsA^m^	1.12 (0.98-1.28)	9.6 × 10^–2^
acoustic_TrailsB^n^	0.69 (0.59-0.81)	*1.0 × 10* ^–5^
acoustic_HVOT^o^	0.91 (0.81-1.03)	1.4 × 10^–1^
acoustic_FAS^p^	0.72 (0.64-0.81)	*3.9 × 10* ^–8^
acoustic_CNT_Animal^q^	0.70 (0.61-0.80)	*2.3 × 10* ^–7^

^a^Significant associations were claimed if *P*<.05/16≈3.1 × 10^–3^.

^b^LMi: Logical Memory—Immediate Recall.

^c^LMd: Logical Memory—Delayed Recall.

^d^LMr: Logical Memory—Recognition.

^e^VRi: Visual Reproduction—Immediate Recall.

^f^VRd: Visual Reproduction—Delayed Recall.

^g^VRr: Visual Reproduction—Recognition.

^h^PASr: Paired Associate Learning—Recognition.

^i^DSf: Digit Span—Forward.

^j^DSb: Digit Span—Backward.

^k^SIM: Similarities.

^l^BNT30: Boston Naming Test—30-item version.

^m^TrailsA: Trail Making Test A.

^n^TrailsB: Trail Making Test B.

^o^HVOT: Hooper Visual Organization Test.

^p^FAS: Controlled Oral Word Association Test.

^q^CNT_Animal: Category Naming Test—Animal.

**Table 6:** Association between acoustic composite scores and incident mild cognitive impairment.

Acoustic composite score	Hazard ratio (95% CI)	*P* value^a^
acoustic_LMi^b^	0.60 (0.47-0.77)	*5.1 × 10* ^–5^
acoustic_LMd^c^	0.76 (0.59-0.97)	2.9 × 10^–2^
acoustic_LMr^d^	0.74 (0.61-0.91)	3.9 × 10^–3^
acoustic_VRi^e^	1.28 (1.10-1.48)	*1.1 × 10* ^–3^
acoustic_VRd^f^	1.25 (1.08-1.44)	*2.4 × 10* ^–3^
acoustic_VRr^g^	0.44 (0.33-0.59)	*6.0 × 10* ^–8^
acoustic_PASr^h^	1.11 (0.95-1.30)	2.0 × 10^–1^
acoustic_DSf^i^	1.11 (0.96-1.29)	1.6 × 10^–1^
acoustic_DSb^j^	1.09 (0.93-1.27)	2.9 × 10^–1^
acoustic_SIM^k^	1.37 (1.16-1.61)	*1.7 × 10* ^–4^
acoustic_BNT30^l^	1.23 (1.06-1.43)	6.4 × 10^–3^
acoustic_TrailsA^m^	0.75 (0.61-0.93)	7.9 × 10^–3^
acoustic_TrailsB^n^	2.03 (1.58-2.60)	*2.5 × 10* ^–8^
acoustic_HVOT^o^	0.78 (0.67-0.91)	*1.7 × 10* ^–3^
acoustic_FAS^p^	0.87 (0.76-1.01)	6.1 × 10^–2^
acoustic_CNT_Animal^q^	0.85 (0.70-1.02)	8.6 × 10^–2^

^a^Significant associations were claimed if *P*<.05/16≈3.1 × 10^–3^.

^b^LMi: Logical Memory—Immediate Recall.

^c^LMd: Logical Memory—Delayed Recall.

^d^LMr: Logical Memory—Recognition.

^e^VRi: Visual Reproduction—Immediate Recall.

^f^VRd: Visual Reproduction—Delayed Recall.

^g^VRr: Visual Reproduction—Recognition.

^h^PASr: Paired Associate Learning—Recognition.

^i^DSf: Digit Span—Forward.

^j^DSb: Digit Span—Backward.

^k^SIM: Similarities.

^l^BNT30: Boston Naming Test—30-item version.

^m^TrailsA: Trail Making Test A.

^n^TrailsB: Trail Making Test B.

^o^HVOT: Hooper Visual Organization Test.

^p^FAS: Controlled Oral Word Association Test.

^q^CNT_Animal: Category Naming Test—Animal.

## Discussion

### Principal Findings

Relating acoustic features with NP test performance is potentially a novel way for screening at the preclinical stages of AD and other dementias. This paper clarifies the relationship between comprehensive acoustic features and NP test performance on large cohort data. Representations relative spectra–style filtered auditory spectrum (spectral), MFCC (cepstral), and magnitude of spectral features (spectral) are 3 categories of acoustic features that were significantly associated with NP test performance. Representations relative spectra–style filtered auditory spectrum is a filtered representation of an audio signal that is robust to additive and convolutional noise [39]. MFCC is a standardized technique for audio feature extraction [40]. It helps in reducing the frequency information of the input speech signal into coefficients, which represent audio based on the perception of human auditory systems. Prior studies have detected changes of these features in people with neurodegenerative processes [41-43]. The acoustic composite score generated for each NP test was a linear combination of LLD features, which are clinically easily interpretable. As stated in the results above, 4 acoustic composite scores were significantly associated with prevalent MCI, and 7 were also found to be significantly associated with incident MCI. Furthermore, the score corresponding to TrailsB test is significantly associated with both prevalent MCI and incident MCI.

Results could expand current evidence regarding the predictive ability of digital voice on MCI that are critical to monitor early cognitive decline. The added predictive ability of acoustic features was evaluated by constructing random forest models with baseline features and additional acoustic composite scores. The model with baseline features and 7 acoustic composite scores corresponding to LMi, VRi, VRd, VRr, SIM, TrailsB, and HVOT tests could achieve an AUC of 0.755 for incident MCI prediction. Monitoring acoustic features outside of the clinical settings offers a more convenient way to aid in the assessment of cognitive health than traditional methods. Increasing evidence suggests that the human voice can be a predictor of cognitive decline before a clinical diagnosis of AD is made [44]. It has been used to screen for MCI [45], dementia [46], and other neurodegenerative diseases such as Parkinson [47] and Huntington disease [48] because of its ease of administration and clinical assessment capability. Moreover, the easy acquisition of voice in daily life makes it an ideal measure for long-term monitoring of cognitive status. However, there is a lack of research about the relationship between acoustic features and NP tests that reflect multiple cognitive functions. Our study could provide some construct validity for this point. In this study, we recorded voice for NP tests that require verbal responses. Although some NP tests do not require verbal responses, these tests might tap some cognitive domains similar to those that require verbal responses. We therefore included these tests as well to capture potential application of acoustic characteristics to assess different cognitive domains. Each NP test might require multiple cognitive domains to complete, which might be shared with other NP tests with subtle differences. Given the rich information from human voice, our study suggests that acoustic features might serve as a new data modality to test this nuance.

Notably, the association between acoustic features and a standard epidemiologic NP test procedure was examined based on participants from a community-based cohort with a diverse range of ages and health conditions. The large volume of voice data provides a more robust representation of participants. Each voice recording lasts, on average, around an hour and contains a wealth of information. The longitudinal collection of data provides a great opportunity to assess the cognitive health of participants throughout the entire course of the disease and prospectively reveals a temporal relationship between acoustic features and MCI. It is worth to noting that 4 of the acoustic composite scores (*acoustic_LMr*, *acoustic_TrailsB*, *acoustic_FAS*, and *acoustic_CNT_Animal*) were significantly associated with prevalent MCI, but 7 acoustic composite scores (*acoustic_LMi*, *acoustic_VRi*, *acoustic_VRd*, *acoustic_VRr*, *acoustic_SIM*, *acoustic_TrailsB*, and *acoustic_HVOT*) were associated with incident MCI. It seems that the voice characteristics differentiating prevalent MCI cases from patients who are still cognitively intact are different from the voice characteristics that are predictive of future risk of cognitive impairment. Future research is needed to further investigate the potential mechanisms that underlie these features and help to account for the MCI prevalence and incidence difference. Further, this study found differences in acoustic features between TrailsA and TrailsB, which provides confirmatory evidence that acoustic features are differential for different cognitive domains. TrailsA, as a measure of simple attention compared to the more complex executive functions measured by TrailsB [49], would be expected to have different acoustic features that would be aligned with motor control and perceptual complexity [50] in the latter and not the former. These differential results suggest that acoustic features might provide a way to detect such subtle differences across cognitive domains. The patterns of acoustic features that are accurately representative of the comprehensive range of cognitive domains will be further explored in future studies.

This study also has some limitations. First, the use of NP tests to diagnose MCI may have led to some circularity and an overestimation of the diagnosis performance [32]. Second, despite that diagnoses are arrived at through a careful adjudication process, there may be some misclassification of MCI. Third, although the FHS collected the voice recordings in a well-controlled environment, there might still be some other factors affecting the quality of voice that were not taken into account. Fourth, this study did not consider linguistic features, which has been shown to be effective in predicting cognitive status. Although we recognize that the inclusion of linguistic features might further improve the prediction of incident MCI, we chose to focus on acoustic features because they are much more generalizable to a broader population, including potentially to other languages. Linguistic features are much more likely to be biased by language, culture, and education. Finally, FHS participants were mostly of European ancestry and English speakers; therefore, the applicability of our findings to populations of another race and ethnicity needs to be examined.

### Conclusion

We examined the association of acoustic features with specific cognitive functions—prevalent and incident MCI—in a large community-based population. Overall, this study’s establishment of a relationship between MCI risk and human voice features provides foundational evidence for an alternative cognitive assessment approach that is cost-effective and easy to administer for detecting cognition-related disorders. Multiple acoustic features were significantly associated with NP test performance and MCI and could be potentially used as a digital biomarker for early cognitive impairment monitoring.

## Appendix

Multimedia Appendix 1 Supplemental tables and figure.

## Abbreviations

AD: Alzheimer disease
AUC: area under the curve
FAS: Controlled Oral Word Association Test
FHS: Framingham Heart Study
HVOT: Hooper Visual Organization Test
LLD: low-level descriptor
LMi: Logical Memory—Immediate Recall
MCI: mild cognitive impairment
MFCC: Mel-frequency cepstral coefficient VRi: Visual Reproduction—Immediate Recall
NP: neuropsychological
OR: odds ratio
ROC: receiver operating characteristic
SIM: Similarities
TrailsB: Trail Making Test B
VRd: Visual Reproduction—Delayed Recall
VRr: Visual Reproduction—Recognition

## Data Availability

The derived acoustic features could be requested through a formal research application to the Framingham Heart Study [51].
