# Supplementary material for: Association Between Acoustic Features and Neuropsychological Test Performance in the Framingham Heart Study: Observational Study
Source: J Med Internet Res. 2022 Dec 22;24(12):e42886. doi: 10.2196/42886 (PMC9816957; doi:10.2196/42886)

**Table S1.** Description of acoustic features used in the current study

| **Index** | **Acoustic feature** | **Category** | **Description** |
| --- | --- | --- | --- |
| 1 | F0final_sma | Prosodic | The fundamental frequency computed from the Cepstrum |
| 2 | voicingFinalUnclipped_sma | Sound quality | The voicing probability of the final fundamental frequency candidate. (Unclipped means that it was not set to zero when it falls below the voicing threshold.) |
| 3 | jitterLocal_sma | Sound quality | The local, or frame-to-frame, variation in frequency from period to period. |
| 4 | jitterDDP_sma | Sound quality | The differential frame-to-frame Jitter (the Jitter of the Jitter) |
| 5 | shimmerLocal_sma | Sound quality | The local variation in amplitude deviations between pitch periods. |
| 6 | logHNR_sma | Sound quality | Log of the Harmonics-to-noise ratio, which indexes the degree of hoarseness/amount of additive noise. |
| 7 | audspec_lengthL1norm_sma | Prosodic | Sum of auditory spectrum (loudness). |
| 8 | audspecRasta_lengthL1norm_sma | Prosodic | Sum of RASTA-style filtered auditory spectrum. |
| 9 | pcm_RMSenergy_sma | Prosodic | Root-mean-square signal-frame energy. |
| 10 | pcm_zcr_sma | Prosodic | Zero-crossing rate of time signal. |
| 11 | audSpec_Rfilt_sma[0] | Spectral | RASTA-style filtered auditory spectrum, band 1 |
| 12 | audSpec_Rfilt_sma[1] | Spectral | RASTA-style filtered auditory spectrum, band 2 |
| 13 | audSpec_Rfilt_sma[2] | Spectral | RASTA-style filtered auditory spectrum, band 3 |
| 14 | audSpec_Rfilt_sma[3] | Spectral | RASTA-style filtered auditory spectrum, band 4 |
| 15 | audSpec_Rfilt_sma[4] | Spectral | RASTA-style filtered auditory spectrum, band 5 |
| 16 | audSpec_Rfilt_sma[5] | Spectral | RASTA-style filtered auditory spectrum, band 6 |
| 17 | audSpec_Rfilt_sma[6] | Spectral | RASTA-style filtered auditory spectrum, band 7 |
| 18 | audSpec_Rfilt_sma[7] | Spectral | RASTA-style filtered auditory spectrum, band 8 |
| 19 | audSpec_Rfilt_sma[8] | Spectral | RASTA-style filtered auditory spectrum, band 9 |
| 20 | audSpec_Rfilt_sma[9] | Spectral | RASTA-style filtered auditory spectrum, band 10 |
| 21 | audSpec_Rfilt_sma[10] | Spectral | RASTA-style filtered auditory spectrum, band 11 |
| 22 | audSpec_Rfilt_sma[11] | Spectral | RASTA-style filtered auditory spectrum, band 12 |
| 23 | audSpec_Rfilt_sma[12] | Spectral | RASTA-style filtered auditory spectrum, band 13 |
| 24 | audSpec_Rfilt_sma[13] | Spectral | RASTA-style filtered auditory spectrum, band 14 |
| 25 | audSpec_Rfilt_sma[14] | Spectral | RASTA-style filtered auditory spectrum, band 15 |
| 26 | audSpec_Rfilt_sma[15] | Spectral | RASTA-style filtered auditory spectrum, band 16 |
| 27 | audSpec_Rfilt_sma[16] | Spectral | RASTA-style filtered auditory spectrum, band 17 |
| 28 | audSpec_Rfilt_sma[17] | Spectral | RASTA-style filtered auditory spectrum, band 18 |
| 29 | audSpec_Rfilt_sma[18] | Spectral | RASTA-style filtered auditory spectrum, band 19 |
| 30 | audSpec_Rfilt_sma[19] | Spectral | RASTA-style filtered auditory spectrum, band 20 |
| 31 | audSpec_Rfilt_sma[20] | Spectral | RASTA-style filtered auditory spectrum, band 21 |
| 32 | audSpec_Rfilt_sma[21] | Spectral | RASTA-style filtered auditory spectrum, band 22 |
| 33 | audSpec_Rfilt_sma[22] | Spectral | RASTA-style filtered auditory spectrum, band 23 |
| 34 | audSpec_Rfilt_sma[23] | Spectral | RASTA-style filtered auditory spectrum, band 24 |
| 35 | audSpec_Rfilt_sma[24] | Spectral | RASTA-style filtered auditory spectrum, band 25 |
| 36 | audSpec_Rfilt_sma[25] | Spectral | RASTA-style filtered auditory spectrum, band 26 |
| 37 | pcm_fftMag_fband250-650_sma | Spectral | Magnitude of frequency band 250-650 Hz. |
| 38 | pcm_fftMag_fband1000-4000_sma | Spectral | Magnitude of frequency band 1-4 kHz (speech frequency band). |
| 39 | pcm_fftMag_spectralRollOff25.0_sma | Spectral | Magnitude of spectral roll off point 25%. |
| 40 | pcm_fftMag_spectralRollOff50.0_sma | Spectral | Magnitude of spectral roll-off point 75% |
| 41 | pcm_fftMag_spectralRollOff75.0_sma | Spectral | Magnitude of spectral roll-off point 50%. |
| 42 | pcm_fftMag_spectralRollOff90.0_sma | Spectral | Magnitude of spectral roll off point 90%. |
| 43 | pcm_fftMag_spectralFlux_sma | Spectral | Magnitude of spectral flux (how quickly the power spectrum of a signal is changing from frame to frame). |
| 44 | pcm_fftMag_spectralCentroid_sma | Spectral | Magnitude of the spectral centroid (the central mass of the audio spectrum). |
| 45 | pcm_fftMag_spectralEntropy_sma | Spectral | Magnitude of spectral entropy (measure of its spectral power distribution). |
| 46 | pcm_fftMag_spectralVariance_sma | Spectral | Magnitude of spectral variance. |
| 47 | pcm_fftMag_spectralSkewness_sma | Spectral | Magnitude of spectral skewness. |
| 48 | pcm_fftMag_spectralKurtosis_sma | Spectral | Magnitude of spectral kurtosis (can indicate the presence of series of transients and their locations in the frequency domain). |
| 49 | pcm_fftMag_spectralSlope_sma | Spectral | Magnitude of spectral slope. |
| 50 | pcm_fftMag_psySharpness_sma | Spectral | Magnitude of psychoacoustic sharpness. |
| 51 | pcm_fftMag_spectralHarmonicity_sma | Spectral | Magnitude of psychoacoustic harmonicity, or spectral regularity. |
| 52 | mfcc_sma[1] | Cepstral | Mel-Frequency cepstral coefficient 1 |
| 53 | mfcc_sma[2] | Cepstral | Mel-Frequency cepstral coefficient 2 |
| 54 | mfcc_sma[3] | Cepstral | Mel-Frequency cepstral coefficient 3 |
| 55 | mfcc_sma[4] | Cepstral | Mel-Frequency cepstral coefficient 4 |
| 56 | mfcc_sma[5] | Cepstral | Mel-Frequency cepstral coefficient 5 |
| 57 | mfcc_sma[6] | Cepstral | Mel-Frequency cepstral coefficient 6 |
| 58 | mfcc_sma[7] | Cepstral | Mel-Frequency cepstral coefficient 7 |
| 59 | mfcc_sma[8] | Cepstral | Mel-Frequency cepstral coefficient 8 |
| 60 | mfcc_sma[9] | Cepstral | Mel-Frequency cepstral coefficient 9 |
| 61 | mfcc_sma[10] | Cepstral | Mel-Frequency cepstral coefficient 10 |
| 62 | mfcc_sma[11] | Cepstral | Mel-Frequency cepstral coefficient 11 |
| 63 | mfcc_sma[12] | Cepstral | Mel-Frequency cepstral coefficient 12 |
| 64 | mfcc_sma[13] | Cepstral | Mel-Frequency cepstral coefficient 13 |
| 65 | mfcc_sma[14] | Cepstral | Mel-Frequency cepstral coefficient 14 |

**Table S2.** Association between acoustic features and NP tests

| **NP test** | **acoustic feature** | **Effect**  **size** | **Standard**  **error** | ***P* value^*^** |
| --- | --- | --- | --- | --- |
| LMi | F0final_sma | 0.03 | 1.04E-02 | 1.05E-02 |
| LMi | voicingFinalUnclipped_sma | 0.04 | 9.32E-03 | 9.47E-05 |
| LMi | jitterLocal_sma | -0.02 | 9.73E-03 | 8.85E-02 |
| LMi | jitterDDP_sma | -0.02 | 9.57E-03 | 6.61E-02 |
| LMi | shimmerLocal_sma | -0.02 | 9.99E-03 | 4.32E-02 |
| LMi | logHNR_sma | -0.01 | 1.01E-02 | 2.39E-01 |
| LMi | audspec_lengthL1norm_sma | 0.03 | 9.57E-03 | 4.64E-03 |
| LMi | audspecRasta_lengthL1norm_sma | 0.00 | 1.05E-02 | 9.86E-01 |
| LMi | pcm_RMSenergy_sma | 0.02 | 9.49E-03 | 3.04E-02 |
| LMi | pcm_zcr_sma | 0.02 | 9.11E-03 | 9.79E-03 |
| LMi | audSpec_Rfilt_sma[0] | 0.02 | 9.90E-03 | 5.47E-02 |
| LMi | audSpec_Rfilt_sma[1] | 0.01 | 1.01E-02 | 2.51E-01 |
| LMi | audSpec_Rfilt_sma[2] | -0.01 | 1.04E-02 | 5.01E-01 |
| LMi | audSpec_Rfilt_sma[3] | -0.01 | 1.05E-02 | 5.45E-01 |
| LMi | audSpec_Rfilt_sma[4] | 0.00 | 1.04E-02 | 8.96E-01 |
| LMi | audSpec_Rfilt_sma[5] | 0.00 | 1.03E-02 | 9.17E-01 |
| LMi | audSpec_Rfilt_sma[6] | 0.00 | 1.02E-02 | 9.30E-01 |
| LMi | audSpec_Rfilt_sma[7] | 0.00 | 1.01E-02 | 9.66E-01 |
| LMi | audSpec_Rfilt_sma[8] | 0.00 | 1.00E-02 | 6.37E-01 |
| LMi | audSpec_Rfilt_sma[9] | -0.01 | 1.00E-02 | 3.77E-01 |
| LMi | audSpec_Rfilt_sma[10] | -0.01 | 1.00E-02 | 1.52E-01 |
| LMi | audSpec_Rfilt_sma[11] | -0.02 | 1.02E-02 | 1.01E-01 |
| LMi | audSpec_Rfilt_sma[12] | -0.02 | 1.03E-02 | 3.80E-02 |
| LMi | audSpec_Rfilt_sma[13] | -0.02 | 1.04E-02 | 7.51E-02 |
| LMi | audSpec_Rfilt_sma[14] | -0.02 | 1.04E-02 | 8.69E-02 |
| LMi | audSpec_Rfilt_sma[15] | -0.02 | 1.03E-02 | 2.11E-02 |
| LMi | audSpec_Rfilt_sma[16] | -0.02 | 1.03E-02 | 8.16E-02 |
| LMi | audSpec_Rfilt_sma[17] | -0.01 | 1.02E-02 | 2.69E-01 |
| LMi | audSpec_Rfilt_sma[18] | -0.01 | 1.02E-02 | 2.33E-01 |
| LMi | audSpec_Rfilt_sma[19] | 0.00 | 9.97E-03 | 7.71E-01 |
| LMi | audSpec_Rfilt_sma[20] | 0.01 | 1.01E-02 | 3.51E-01 |
| LMi | audSpec_Rfilt_sma[21] | 0.01 | 1.03E-02 | 2.53E-01 |
| LMi | audSpec_Rfilt_sma[22] | 0.03 | 1.00E-02 | 3.39E-03 |
| LMi | audSpec_Rfilt_sma[23] | 0.04 | 9.69E-03 | 1.91E-05 |
| LMi | audSpec_Rfilt_sma[24] | 0.05 | 9.51E-03 | 2.01E-06 |
| LMi | audSpec_Rfilt_sma[25] | 0.05 | 9.53E-03 | 2.68E-07 |
| LMi | pcm_fftMag_fband250-650_sma | 0.01 | 9.45E-03 | 2.51E-01 |
| LMi | pcm_fftMag_fband1000-4000_sma | 0.02 | 9.64E-03 | 1.05E-02 |
| LMi | pcm_fftMag_spectralRollOff25.0_sma | 0.03 | 9.18E-03 | 2.11E-03 |
| LMi | pcm_fftMag_spectralRollOff50.0_sma | 0.03 | 9.16E-03 | 5.93E-03 |
| LMi | pcm_fftMag_spectralRollOff75.0_sma | 0.02 | 9.16E-03 | 8.20E-03 |
| LMi | pcm_fftMag_spectralRollOff90.0_sma | 0.02 | 9.20E-03 | 1.13E-02 |
| LMi | pcm_fftMag_spectralFlux_sma | 0.02 | 9.54E-03 | 1.43E-02 |
| LMi | pcm_fftMag_spectralCentroid_sma | 0.02 | 9.15E-03 | 6.72E-03 |
| LMi | pcm_fftMag_spectralEntropy_sma | 0.03 | 9.22E-03 | 5.45E-03 |
| LMi | pcm_fftMag_spectralVariance_sma | 0.02 | 9.13E-03 | 3.56E-02 |
| LMi | pcm_fftMag_spectralSkewness_sma | -0.03 | 8.93E-03 | 7.07E-04 |
| LMi | pcm_fftMag_spectralKurtosis_sma | -0.02 | 8.97E-03 | 2.52E-02 |
| LMi | pcm_fftMag_spectralSlope_sma | -0.02 | 9.36E-03 | 6.51E-02 |
| LMi | pcm_fftMag_psySharpness_sma | 0.03 | 9.19E-03 | 4.35E-03 |
| LMi | pcm_fftMag_spectralHarmonicity_sma | 0.02 | 9.48E-03 | 3.64E-02 |
| LMi | mfcc_sma[1] | -0.01 | 9.18E-03 | 1.80E-01 |
| LMi | mfcc_sma[2] | -0.03 | 9.18E-03 | 4.58E-03 |
| LMi | mfcc_sma[3] | -0.01 | 9.78E-03 | 4.51E-01 |
| LMi | mfcc_sma[4] | -0.04 | 9.39E-03 | 1.91E-04 |
| LMi | mfcc_sma[5] | 0.01 | 9.41E-03 | 4.25E-01 |
| LMi | mfcc_sma[6] | -0.02 | 9.35E-03 | 1.17E-02 |
| LMi | mfcc_sma[7] | -0.01 | 9.01E-03 | 2.26E-01 |
| LMi | mfcc_sma[8] | -0.01 | 9.39E-03 | 1.23E-01 |
| LMi | mfcc_sma[9] | -0.02 | 9.63E-03 | 1.23E-02 |
| LMi | mfcc_sma[10] | -0.01 | 9.06E-03 | 1.25E-01 |
| LMi | mfcc_sma[11] | -0.02 | 9.15E-03 | 9.04E-02 |
| LMi | mfcc_sma[12] | -0.01 | 9.14E-03 | 2.14E-01 |
| LMi | mfcc_sma[13] | -0.03 | 9.49E-03 | 3.66E-03 |
| LMi | mfcc_sma[14] | -0.04 | 9.57E-03 | 1.03E-04 |
|  |  |  |  |  |
| **LMd** | F0final_sma | 0.02 | 1.03E-02 | 7.89E-02 |
| LMd | voicingFinalUnclipped_sma | 0.02 | 9.23E-03 | 2.08E-02 |
| LMd | jitterLocal_sma | -0.02 | 9.63E-03 | 8.00E-02 |
| LMd | jitterDDP_sma | -0.02 | 9.48E-03 | 5.01E-02 |
| LMd | shimmerLocal_sma | -0.02 | 9.90E-03 | 4.66E-02 |
| LMd | logHNR_sma | -0.01 | 9.97E-03 | 1.63E-01 |
| LMd | audspec_lengthL1norm_sma | 0.02 | 9.46E-03 | 4.62E-02 |
| LMd | audspecRasta_lengthL1norm_sma | 0.01 | 1.04E-02 | 4.39E-01 |
| LMd | pcm_RMSenergy_sma | 0.01 | 9.38E-03 | 1.40E-01 |
| LMd | pcm_zcr_sma | 0.01 | 9.01E-03 | 1.13E-01 |
| LMd | audSpec_Rfilt_sma[0] | 0.01 | 9.81E-03 | 3.47E-01 |
| LMd | audSpec_Rfilt_sma[1] | 0.01 | 1.00E-02 | 5.74E-01 |
| LMd | audSpec_Rfilt_sma[2] | -0.01 | 1.03E-02 | 5.16E-01 |
| LMd | audSpec_Rfilt_sma[3] | 0.00 | 1.03E-02 | 8.42E-01 |
| LMd | audSpec_Rfilt_sma[4] | 0.00 | 1.03E-02 | 7.37E-01 |
| LMd | audSpec_Rfilt_sma[5] | 0.00 | 1.02E-02 | 7.10E-01 |
| LMd | audSpec_Rfilt_sma[6] | 0.00 | 1.01E-02 | 6.39E-01 |
| LMd | audSpec_Rfilt_sma[7] | 0.00 | 1.00E-02 | 6.75E-01 |
| LMd | audSpec_Rfilt_sma[8] | 0.00 | 9.89E-03 | 8.49E-01 |
| LMd | audSpec_Rfilt_sma[9] | 0.00 | 9.91E-03 | 9.90E-01 |
| LMd | audSpec_Rfilt_sma[10] | 0.00 | 9.92E-03 | 6.24E-01 |
| LMd | audSpec_Rfilt_sma[11] | -0.01 | 1.00E-02 | 4.99E-01 |
| LMd | audSpec_Rfilt_sma[12] | -0.01 | 1.01E-02 | 3.14E-01 |
| LMd | audSpec_Rfilt_sma[13] | -0.01 | 1.03E-02 | 4.91E-01 |
| LMd | audSpec_Rfilt_sma[14] | -0.01 | 1.03E-02 | 5.22E-01 |
| LMd | audSpec_Rfilt_sma[15] | -0.01 | 1.02E-02 | 3.40E-01 |
| LMd | audSpec_Rfilt_sma[16] | 0.00 | 1.02E-02 | 6.35E-01 |
| LMd | audSpec_Rfilt_sma[17] | 0.00 | 1.01E-02 | 8.98E-01 |
| LMd | audSpec_Rfilt_sma[18] | 0.00 | 1.00E-02 | 9.83E-01 |
| LMd | audSpec_Rfilt_sma[19] | 0.01 | 9.86E-03 | 4.34E-01 |
| LMd | audSpec_Rfilt_sma[20] | 0.02 | 9.95E-03 | 6.85E-02 |
| LMd | audSpec_Rfilt_sma[21] | 0.02 | 1.02E-02 | 5.37E-02 |
| LMd | audSpec_Rfilt_sma[22] | 0.03 | 9.88E-03 | 1.46E-03 |
| LMd | audSpec_Rfilt_sma[23] | 0.04 | 9.57E-03 | 1.76E-04 |
| LMd | audSpec_Rfilt_sma[24] | 0.04 | 9.40E-03 | 1.03E-04 |
| LMd | audSpec_Rfilt_sma[25] | 0.04 | 9.41E-03 | 1.94E-05 |
| LMd | pcm_fftMag_fband250-650_sma | 0.01 | 9.32E-03 | 4.79E-01 |
| LMd | pcm_fftMag_fband1000-4000_sma | 0.02 | 9.53E-03 | 5.21E-02 |
| LMd | pcm_fftMag_spectralRollOff25.0_sma | 0.02 | 9.08E-03 | 9.32E-02 |
| LMd | pcm_fftMag_spectralRollOff50.0_sma | 0.01 | 9.06E-03 | 1.51E-01 |
| LMd | pcm_fftMag_spectralRollOff75.0_sma | 0.01 | 9.05E-03 | 1.59E-01 |
| LMd | pcm_fftMag_spectralRollOff90.0_sma | 0.01 | 9.09E-03 | 1.56E-01 |
| LMd | pcm_fftMag_spectralFlux_sma | 0.02 | 9.43E-03 | 9.01E-02 |
| LMd | pcm_fftMag_spectralCentroid_sma | 0.01 | 9.05E-03 | 1.26E-01 |
| LMd | pcm_fftMag_spectralEntropy_sma | 0.02 | 9.10E-03 | 8.65E-02 |
| LMd | pcm_fftMag_spectralVariance_sma | 0.01 | 9.02E-03 | 1.80E-01 |
| LMd | pcm_fftMag_spectralSkewness_sma | -0.02 | 8.83E-03 | 1.46E-02 |
| LMd | pcm_fftMag_spectralKurtosis_sma | -0.02 | 8.87E-03 | 4.75E-02 |
| LMd | pcm_fftMag_spectralSlope_sma | -0.01 | 9.24E-03 | 2.25E-01 |
| LMd | pcm_fftMag_psySharpness_sma | 0.01 | 9.08E-03 | 9.88E-02 |
| LMd | pcm_fftMag_spectralHarmonicity_sma | 0.01 | 9.35E-03 | 1.38E-01 |
| LMd | mfcc_sma[1] | -0.01 | 9.08E-03 | 2.13E-01 |
| LMd | mfcc_sma[2] | -0.01 | 9.08E-03 | 2.26E-01 |
| LMd | mfcc_sma[3] | -0.01 | 9.67E-03 | 1.93E-01 |
| LMd | mfcc_sma[4] | -0.02 | 9.28E-03 | 3.43E-02 |
| LMd | mfcc_sma[5] | 0.00 | 9.31E-03 | 7.39E-01 |
| LMd | mfcc_sma[6] | -0.02 | 9.25E-03 | 4.09E-02 |
| LMd | mfcc_sma[7] | 0.00 | 8.91E-03 | 8.15E-01 |
| LMd | mfcc_sma[8] | -0.01 | 9.29E-03 | 1.67E-01 |
| LMd | mfcc_sma[9] | -0.02 | 9.56E-03 | 9.84E-02 |
| LMd | mfcc_sma[10] | 0.00 | 8.95E-03 | 6.31E-01 |
| LMd | mfcc_sma[11] | -0.02 | 9.05E-03 | 9.06E-02 |
| LMd | mfcc_sma[12] | 0.00 | 9.06E-03 | 9.95E-01 |
| LMd | mfcc_sma[13] | -0.02 | 9.41E-03 | 7.25E-02 |
| LMd | mfcc_sma[14] | -0.03 | 9.48E-03 | 1.26E-03 |
|  |  |  |  |  |
| **LMr** | F0final_sma | 0.00 | 1.16E-02 | 7.67E-01 |
| LMr | voicingFinalUnclipped_sma | 0.02 | 1.05E-02 | 3.67E-02 |
| LMr | jitterLocal_sma | -0.02 | 1.09E-02 | 3.54E-02 |
| LMr | jitterDDP_sma | -0.02 | 1.08E-02 | 3.42E-02 |
| LMr | shimmerLocal_sma | -0.03 | 1.12E-02 | 6.02E-03 |
| LMr | logHNR_sma | -0.03 | 1.13E-02 | 1.12E-02 |
| LMr | audspec_lengthL1norm_sma | 0.01 | 1.07E-02 | 4.54E-01 |
| LMr | audspecRasta_lengthL1norm_sma | 0.02 | 1.18E-02 | 3.92E-02 |
| LMr | pcm_RMSenergy_sma | 0.01 | 1.06E-02 | 4.26E-01 |
| LMr | pcm_zcr_sma | 0.00 | 1.04E-02 | 9.16E-01 |
| LMr | audSpec_Rfilt_sma[0] | 0.00 | 1.10E-02 | 9.90E-01 |
| LMr | audSpec_Rfilt_sma[1] | 0.00 | 1.12E-02 | 8.89E-01 |
| LMr | audSpec_Rfilt_sma[2] | 0.00 | 1.16E-02 | 7.60E-01 |
| LMr | audSpec_Rfilt_sma[3] | 0.01 | 1.17E-02 | 6.08E-01 |
| LMr | audSpec_Rfilt_sma[4] | 0.01 | 1.16E-02 | 4.28E-01 |
| LMr | audSpec_Rfilt_sma[5] | 0.01 | 1.16E-02 | 2.95E-01 |
| LMr | audSpec_Rfilt_sma[6] | 0.02 | 1.15E-02 | 1.76E-01 |
| LMr | audSpec_Rfilt_sma[7] | 0.02 | 1.14E-02 | 1.58E-01 |
| LMr | audSpec_Rfilt_sma[8] | 0.02 | 1.13E-02 | 1.35E-01 |
| LMr | audSpec_Rfilt_sma[9] | 0.02 | 1.13E-02 | 1.01E-01 |
| LMr | audSpec_Rfilt_sma[10] | 0.02 | 1.13E-02 | 1.40E-01 |
| LMr | audSpec_Rfilt_sma[11] | 0.01 | 1.15E-02 | 2.29E-01 |
| LMr | audSpec_Rfilt_sma[12] | 0.01 | 1.16E-02 | 2.99E-01 |
| LMr | audSpec_Rfilt_sma[13] | 0.01 | 1.17E-02 | 2.29E-01 |
| LMr | audSpec_Rfilt_sma[14] | 0.01 | 1.17E-02 | 2.58E-01 |
| LMr | audSpec_Rfilt_sma[15] | 0.01 | 1.16E-02 | 2.78E-01 |
| LMr | audSpec_Rfilt_sma[16] | 0.01 | 1.15E-02 | 2.15E-01 |
| LMr | audSpec_Rfilt_sma[17] | 0.02 | 1.15E-02 | 1.38E-01 |
| LMr | audSpec_Rfilt_sma[18] | 0.01 | 1.14E-02 | 2.33E-01 |
| LMr | audSpec_Rfilt_sma[19] | 0.02 | 1.12E-02 | 1.37E-01 |
| LMr | audSpec_Rfilt_sma[20] | 0.02 | 1.13E-02 | 5.06E-02 |
| LMr | audSpec_Rfilt_sma[21] | 0.02 | 1.15E-02 | 4.02E-02 |
| LMr | audSpec_Rfilt_sma[22] | 0.04 | 1.11E-02 | 9.27E-04 |
| LMr | audSpec_Rfilt_sma[23] | 0.04 | 1.08E-02 | 2.31E-04 |
| LMr | audSpec_Rfilt_sma[24] | 0.04 | 1.06E-02 | 6.23E-04 |
| LMr | audSpec_Rfilt_sma[25] | 0.04 | 1.06E-02 | 4.40E-04 |
| LMr | pcm_fftMag_fband250-650_sma | 0.00 | 1.05E-02 | 7.75E-01 |
| LMr | pcm_fftMag_fband1000-4000_sma | 0.01 | 1.07E-02 | 4.67E-01 |
| LMr | pcm_fftMag_spectralRollOff25.0_sma | 0.00 | 1.04E-02 | 8.79E-01 |
| LMr | pcm_fftMag_spectralRollOff50.0_sma | 0.00 | 1.04E-02 | 9.61E-01 |
| LMr | pcm_fftMag_spectralRollOff75.0_sma | 0.00 | 1.04E-02 | 9.22E-01 |
| LMr | pcm_fftMag_spectralRollOff90.0_sma | 0.00 | 1.05E-02 | 8.00E-01 |
| LMr | pcm_fftMag_spectralFlux_sma | 0.01 | 1.06E-02 | 3.63E-01 |
| LMr | pcm_fftMag_spectralCentroid_sma | 0.00 | 1.04E-02 | 9.33E-01 |
| LMr | pcm_fftMag_spectralEntropy_sma | 0.00 | 1.05E-02 | 7.23E-01 |
| LMr | pcm_fftMag_spectralVariance_sma | 0.00 | 1.04E-02 | 8.72E-01 |
| LMr | pcm_fftMag_spectralSkewness_sma | 0.00 | 1.02E-02 | 8.24E-01 |
| LMr | pcm_fftMag_spectralKurtosis_sma | 0.00 | 1.03E-02 | 8.80E-01 |
| LMr | pcm_fftMag_spectralSlope_sma | -0.01 | 1.05E-02 | 4.80E-01 |
| LMr | pcm_fftMag_psySharpness_sma | 0.00 | 1.05E-02 | 9.16E-01 |
| LMr | pcm_fftMag_spectralHarmonicity_sma | 0.01 | 1.05E-02 | 5.03E-01 |
| LMr | mfcc_sma[1] | 0.00 | 1.04E-02 | 7.34E-01 |
| LMr | mfcc_sma[2] | 0.00 | 1.04E-02 | 6.61E-01 |
| LMr | mfcc_sma[3] | 0.01 | 1.10E-02 | 5.28E-01 |
| LMr | mfcc_sma[4] | -0.02 | 1.06E-02 | 1.17E-01 |
| LMr | mfcc_sma[5] | 0.02 | 1.06E-02 | 1.34E-01 |
| LMr | mfcc_sma[6] | -0.02 | 1.06E-02 | 1.23E-01 |
| LMr | mfcc_sma[7] | 0.00 | 1.02E-02 | 8.56E-01 |
| LMr | mfcc_sma[8] | 0.00 | 1.06E-02 | 6.83E-01 |
| LMr | mfcc_sma[9] | -0.02 | 1.08E-02 | 5.73E-02 |
| LMr | mfcc_sma[10] | 0.01 | 1.02E-02 | 6.04E-01 |
| LMr | mfcc_sma[11] | -0.01 | 1.04E-02 | 3.62E-01 |
| LMr | mfcc_sma[12] | 0.00 | 1.03E-02 | 8.59E-01 |
| LMr | mfcc_sma[13] | 0.00 | 1.06E-02 | 8.51E-01 |
| LMr | mfcc_sma[14] | -0.02 | 1.07E-02 | 1.53E-01 |
|  |  |  |  |  |
| **VRi** | F0final_sma | 0.00 | 9.67E-03 | 7.27E-01 |
| VRi | voicingFinalUnclipped_sma | 0.04 | 8.60E-03 | 7.54E-07 |
| VRi | jitterLocal_sma | 0.05 | 9.07E-03 | 3.28E-07 |
| VRi | jitterDDP_sma | 0.04 | 8.95E-03 | 5.23E-05 |
| VRi | shimmerLocal_sma | 0.05 | 9.25E-03 | 3.20E-08 |
| VRi | logHNR_sma | 0.02 | 9.32E-03 | 3.15E-02 |
| VRi | audspec_lengthL1norm_sma | -0.06 | 8.84E-03 | 8.51E-11 |
| VRi | audspecRasta_lengthL1norm_sma | 0.07 | 9.73E-03 | 1.93E-11 |
| VRi | pcm_RMSenergy_sma | 0.00 | 8.88E-03 | 5.86E-01 |
| VRi | pcm_zcr_sma | -0.13 | 8.23E-03 | 1.24E-55 |
| VRi | audSpec_Rfilt_sma[0] | 0.03 | 9.25E-03 | 8.60E-04 |
| VRi | audSpec_Rfilt_sma[1] | 0.01 | 9.56E-03 | 5.04E-01 |
| VRi | audSpec_Rfilt_sma[2] | 0.03 | 9.81E-03 | 8.21E-04 |
| VRi | audSpec_Rfilt_sma[3] | 0.07 | 9.75E-03 | 2.26E-14 |
| VRi | audSpec_Rfilt_sma[4] | 0.09 | 9.57E-03 | 6.46E-23 |
| VRi | audSpec_Rfilt_sma[5] | 0.11 | 9.41E-03 | 1.37E-31 |
| VRi | audSpec_Rfilt_sma[6] | 0.11 | 9.37E-03 | 7.96E-32 |
| VRi | audSpec_Rfilt_sma[7] | 0.10 | 9.27E-03 | 7.12E-28 |
| VRi | audSpec_Rfilt_sma[8] | 0.10 | 9.14E-03 | 8.01E-30 |
| VRi | audSpec_Rfilt_sma[9] | 0.11 | 9.15E-03 | 5.23E-34 |
| VRi | audSpec_Rfilt_sma[10] | 0.12 | 9.15E-03 | 1.69E-36 |
| VRi | audSpec_Rfilt_sma[11] | 0.11 | 9.29E-03 | 1.89E-30 |
| VRi | audSpec_Rfilt_sma[12] | 0.09 | 9.44E-03 | 7.00E-22 |
| VRi | audSpec_Rfilt_sma[13] | 0.07 | 9.63E-03 | 6.97E-15 |
| VRi | audSpec_Rfilt_sma[14] | 0.07 | 9.62E-03 | 2.55E-12 |
| VRi | audSpec_Rfilt_sma[15] | 0.05 | 9.56E-03 | 4.97E-08 |
| VRi | audSpec_Rfilt_sma[16] | 0.02 | 9.51E-03 | 4.43E-02 |
| VRi | audSpec_Rfilt_sma[17] | 0.01 | 9.48E-03 | 1.53E-01 |
| VRi | audSpec_Rfilt_sma[18] | 0.01 | 9.42E-03 | 1.17E-01 |
| VRi | audSpec_Rfilt_sma[19] | 0.01 | 9.26E-03 | 3.92E-01 |
| VRi | audSpec_Rfilt_sma[20] | 0.00 | 9.35E-03 | 5.94E-01 |
| VRi | audSpec_Rfilt_sma[21] | 0.04 | 9.57E-03 | 1.51E-05 |
| VRi | audSpec_Rfilt_sma[22] | 0.01 | 9.30E-03 | 5.42E-01 |
| VRi | audSpec_Rfilt_sma[23] | -0.04 | 8.98E-03 | 6.12E-05 |
| VRi | audSpec_Rfilt_sma[24] | -0.06 | 8.77E-03 | 1.77E-12 |
| VRi | audSpec_Rfilt_sma[25] | -0.05 | 8.82E-03 | 7.75E-09 |
| VRi | pcm_fftMag_fband250-650_sma | 0.06 | 8.78E-03 | 1.29E-12 |
| VRi | pcm_fftMag_fband1000-4000_sma | -0.05 | 8.92E-03 | 6.16E-09 |
| VRi | pcm_fftMag_spectralRollOff25.0_sma | -0.11 | 8.36E-03 | 1.01E-39 |
| VRi | pcm_fftMag_spectralRollOff50.0_sma | -0.12 | 8.31E-03 | 1.42E-47 |
| VRi | pcm_fftMag_spectralRollOff75.0_sma | -0.13 | 8.29E-03 | 2.80E-52 |
| VRi | pcm_fftMag_spectralRollOff90.0_sma | -0.13 | 8.31E-03 | 4.62E-56 |
| VRi | pcm_fftMag_spectralFlux_sma | -0.01 | 8.92E-03 | 2.53E-01 |
| VRi | pcm_fftMag_spectralCentroid_sma | -0.13 | 8.28E-03 | 1.57E-52 |
| VRi | pcm_fftMag_spectralEntropy_sma | -0.12 | 8.33E-03 | 1.04E-50 |
| VRi | pcm_fftMag_spectralVariance_sma | -0.13 | 8.26E-03 | 3.81E-56 |
| VRi | pcm_fftMag_spectralSkewness_sma | 0.08 | 8.24E-03 | 1.14E-24 |
| VRi | pcm_fftMag_spectralKurtosis_sma | 0.05 | 8.96E-03 | 4.55E-07 |
| VRi | pcm_fftMag_spectralSlope_sma | -0.04 | 9.69E-03 | 4.30E-06 |
| VRi | pcm_fftMag_psySharpness_sma | -0.12 | 8.32E-03 | 1.60E-50 |
| VRi | pcm_fftMag_spectralHarmonicity_sma | 0.02 | 8.83E-03 | 2.57E-02 |
| VRi | mfcc_sma[1] | 0.14 | 8.28E-03 | 1.47E-65 |
| VRi | mfcc_sma[2] | 0.05 | 8.46E-03 | 8.59E-08 |
| VRi | mfcc_sma[3] | 0.13 | 8.90E-03 | 3.07E-45 |
| VRi | mfcc_sma[4] | 0.10 | 8.56E-03 | 5.83E-30 |
| VRi | mfcc_sma[5] | -0.12 | 8.55E-03 | 2.13E-44 |
| VRi | mfcc_sma[6] | 0.13 | 8.45E-03 | 2.18E-56 |
| VRi | mfcc_sma[7] | -0.13 | 8.17E-03 | 2.96E-53 |
| VRi | mfcc_sma[8] | 0.12 | 8.52E-03 | 5.53E-42 |
| VRi | mfcc_sma[9] | 0.01 | 8.92E-03 | 1.78E-01 |
| VRi | mfcc_sma[10] | -0.12 | 8.24E-03 | 1.10E-44 |
| VRi | mfcc_sma[11] | 0.14 | 8.23E-03 | 8.41E-66 |
| VRi | mfcc_sma[12] | -0.09 | 8.37E-03 | 5.91E-26 |
| VRi | mfcc_sma[13] | 0.02 | 8.79E-03 | 6.76E-03 |
| VRi | mfcc_sma[14] | 0.08 | 8.83E-03 | 4.88E-21 |
|  |  |  |  |  |
| **VRd** | F0final_sma | -0.01 | 9.56E-03 | 1.85E-01 |
| VRd | voicingFinalUnclipped_sma | 0.03 | 8.43E-03 | 1.93E-03 |
| VRd | jitterLocal_sma | 0.02 | 8.96E-03 | 6.99E-03 |
| VRd | jitterDDP_sma | 0.02 | 8.85E-03 | 4.64E-02 |
| VRd | shimmerLocal_sma | 0.03 | 9.13E-03 | 1.09E-03 |
| VRd | logHNR_sma | 0.01 | 9.19E-03 | 4.58E-01 |
| VRd | audspec_lengthL1norm_sma | -0.05 | 8.68E-03 | 7.31E-09 |
| VRd | audspecRasta_lengthL1norm_sma | 0.05 | 9.60E-03 | 7.27E-07 |
| VRd | pcm_RMSenergy_sma | 0.00 | 8.74E-03 | 9.71E-01 |
| VRd | pcm_zcr_sma | -0.11 | 8.14E-03 | 7.26E-39 |
| VRd | audSpec_Rfilt_sma[0] | 0.01 | 9.14E-03 | 2.04E-01 |
| VRd | audSpec_Rfilt_sma[1] | 0.00 | 9.44E-03 | 7.98E-01 |
| VRd | audSpec_Rfilt_sma[2] | 0.02 | 9.66E-03 | 2.81E-02 |
| VRd | audSpec_Rfilt_sma[3] | 0.05 | 9.60E-03 | 1.25E-08 |
| VRd | audSpec_Rfilt_sma[4] | 0.07 | 9.43E-03 | 8.76E-14 |
| VRd | audSpec_Rfilt_sma[5] | 0.08 | 9.29E-03 | 4.02E-18 |
| VRd | audSpec_Rfilt_sma[6] | 0.08 | 9.24E-03 | 8.44E-19 |
| VRd | audSpec_Rfilt_sma[7] | 0.07 | 9.15E-03 | 3.12E-16 |
| VRd | audSpec_Rfilt_sma[8] | 0.08 | 9.02E-03 | 3.84E-17 |
| VRd | audSpec_Rfilt_sma[9] | 0.08 | 9.03E-03 | 2.37E-20 |
| VRd | audSpec_Rfilt_sma[10] | 0.09 | 9.04E-03 | 6.54E-22 |
| VRd | audSpec_Rfilt_sma[11] | 0.08 | 9.17E-03 | 1.89E-18 |
| VRd | audSpec_Rfilt_sma[12] | 0.07 | 9.31E-03 | 1.87E-13 |
| VRd | audSpec_Rfilt_sma[13] | 0.05 | 9.50E-03 | 7.82E-09 |
| VRd | audSpec_Rfilt_sma[14] | 0.05 | 9.49E-03 | 2.90E-07 |
| VRd | audSpec_Rfilt_sma[15] | 0.04 | 9.43E-03 | 3.40E-05 |
| VRd | audSpec_Rfilt_sma[16] | 0.01 | 9.40E-03 | 1.38E-01 |
| VRd | audSpec_Rfilt_sma[17] | 0.01 | 9.39E-03 | 4.71E-01 |
| VRd | audSpec_Rfilt_sma[18] | 0.01 | 9.32E-03 | 5.52E-01 |
| VRd | audSpec_Rfilt_sma[19] | 0.00 | 9.17E-03 | 8.68E-01 |
| VRd | audSpec_Rfilt_sma[20] | 0.00 | 9.27E-03 | 7.80E-01 |
| VRd | audSpec_Rfilt_sma[21] | 0.03 | 9.45E-03 | 7.70E-04 |
| VRd | audSpec_Rfilt_sma[22] | 0.01 | 9.18E-03 | 5.63E-01 |
| VRd | audSpec_Rfilt_sma[23] | -0.03 | 8.87E-03 | 1.98E-03 |
| VRd | audSpec_Rfilt_sma[24] | -0.05 | 8.67E-03 | 1.66E-08 |
| VRd | audSpec_Rfilt_sma[25] | -0.04 | 8.70E-03 | 7.98E-07 |
| VRd | pcm_fftMag_fband250-650_sma | 0.05 | 8.67E-03 | 3.44E-08 |
| VRd | pcm_fftMag_fband1000-4000_sma | -0.05 | 8.79E-03 | 1.82E-07 |
| VRd | pcm_fftMag_spectralRollOff25.0_sma | -0.09 | 8.26E-03 | 2.55E-30 |
| VRd | pcm_fftMag_spectralRollOff50.0_sma | -0.10 | 8.22E-03 | 6.51E-35 |
| VRd | pcm_fftMag_spectralRollOff75.0_sma | -0.11 | 8.20E-03 | 1.11E-37 |
| VRd | pcm_fftMag_spectralRollOff90.0_sma | -0.11 | 8.21E-03 | 6.07E-39 |
| VRd | pcm_fftMag_spectralFlux_sma | -0.01 | 8.77E-03 | 1.83E-01 |
| VRd | pcm_fftMag_spectralCentroid_sma | -0.10 | 8.19E-03 | 2.05E-37 |
| VRd | pcm_fftMag_spectralEntropy_sma | -0.10 | 8.23E-03 | 2.82E-35 |
| VRd | pcm_fftMag_spectralVariance_sma | -0.11 | 8.17E-03 | 2.62E-38 |
| VRd | pcm_fftMag_spectralSkewness_sma | 0.07 | 8.11E-03 | 1.27E-17 |
| VRd | pcm_fftMag_spectralKurtosis_sma | 0.04 | 8.79E-03 | 1.38E-05 |
| VRd | pcm_fftMag_spectralSlope_sma | -0.03 | 9.55E-03 | 5.32E-04 |
| VRd | pcm_fftMag_psySharpness_sma | -0.10 | 8.22E-03 | 3.31E-36 |
| VRd | pcm_fftMag_spectralHarmonicity_sma | 0.01 | 8.71E-03 | 1.73E-01 |
| VRd | mfcc_sma[1] | 0.11 | 8.22E-03 | 1.01E-42 |
| VRd | mfcc_sma[2] | 0.04 | 8.30E-03 | 2.81E-06 |
| VRd | mfcc_sma[3] | 0.10 | 8.79E-03 | 7.31E-32 |
| VRd | mfcc_sma[4] | 0.08 | 8.43E-03 | 1.77E-21 |
| VRd | mfcc_sma[5] | -0.09 | 8.45E-03 | 3.73E-28 |
| VRd | mfcc_sma[6] | 0.11 | 8.36E-03 | 4.13E-40 |
| VRd | mfcc_sma[7] | -0.10 | 8.08E-03 | 2.03E-32 |
| VRd | mfcc_sma[8] | 0.09 | 8.42E-03 | 7.48E-29 |
| VRd | mfcc_sma[9] | 0.01 | 8.79E-03 | 3.02E-01 |
| VRd | mfcc_sma[10] | -0.09 | 8.14E-03 | 2.49E-27 |
| VRd | mfcc_sma[11] | 0.11 | 8.16E-03 | 3.68E-44 |
| VRd | mfcc_sma[12] | -0.07 | 8.26E-03 | 2.67E-15 |
| VRd | mfcc_sma[13] | 0.02 | 8.64E-03 | 6.14E-02 |
| VRd | mfcc_sma[14] | 0.07 | 8.71E-03 | 5.87E-16 |
|  |  |  |  |  |
| **VRr** | F0final_sma | -0.01 | 1.07E-02 | 2.25E-01 |
| VRr | voicingFinalUnclipped_sma | -0.01 | 9.60E-03 | 4.96E-01 |
| VRr | jitterLocal_sma | 0.01 | 1.01E-02 | 2.08E-01 |
| VRr | jitterDDP_sma | 0.01 | 9.92E-03 | 2.21E-01 |
| VRr | shimmerLocal_sma | 0.01 | 1.03E-02 | 2.96E-01 |
| VRr | logHNR_sma | 0.00 | 1.03E-02 | 8.20E-01 |
| VRr | audspec_lengthL1norm_sma | -0.02 | 9.81E-03 | 1.68E-02 |
| VRr | audspecRasta_lengthL1norm_sma | 0.01 | 1.09E-02 | 3.78E-01 |
| VRr | pcm_RMSenergy_sma | -0.01 | 9.81E-03 | 1.76E-01 |
| VRr | pcm_zcr_sma | -0.03 | 9.51E-03 | 3.19E-04 |
| VRr | audSpec_Rfilt_sma[0] | -0.02 | 1.02E-02 | 3.25E-02 |
| VRr | audSpec_Rfilt_sma[1] | -0.02 | 1.06E-02 | 1.07E-01 |
| VRr | audSpec_Rfilt_sma[2] | -0.01 | 1.09E-02 | 4.42E-01 |
| VRr | audSpec_Rfilt_sma[3] | 0.00 | 1.09E-02 | 9.96E-01 |
| VRr | audSpec_Rfilt_sma[4] | 0.01 | 1.07E-02 | 5.22E-01 |
| VRr | audSpec_Rfilt_sma[5] | 0.01 | 1.06E-02 | 4.01E-01 |
| VRr | audSpec_Rfilt_sma[6] | 0.01 | 1.06E-02 | 1.68E-01 |
| VRr | audSpec_Rfilt_sma[7] | 0.02 | 1.05E-02 | 1.52E-01 |
| VRr | audSpec_Rfilt_sma[8] | 0.02 | 1.04E-02 | 1.01E-01 |
| VRr | audSpec_Rfilt_sma[9] | 0.02 | 1.04E-02 | 6.89E-02 |
| VRr | audSpec_Rfilt_sma[10] | 0.02 | 1.04E-02 | 7.85E-02 |
| VRr | audSpec_Rfilt_sma[11] | 0.01 | 1.05E-02 | 1.95E-01 |
| VRr | audSpec_Rfilt_sma[12] | 0.01 | 1.06E-02 | 2.06E-01 |
| VRr | audSpec_Rfilt_sma[13] | 0.01 | 1.08E-02 | 2.29E-01 |
| VRr | audSpec_Rfilt_sma[14] | 0.01 | 1.08E-02 | 1.90E-01 |
| VRr | audSpec_Rfilt_sma[15] | 0.01 | 1.07E-02 | 1.71E-01 |
| VRr | audSpec_Rfilt_sma[16] | 0.01 | 1.06E-02 | 3.87E-01 |
| VRr | audSpec_Rfilt_sma[17] | 0.01 | 1.06E-02 | 5.74E-01 |
| VRr | audSpec_Rfilt_sma[18] | 0.00 | 1.05E-02 | 7.55E-01 |
| VRr | audSpec_Rfilt_sma[19] | 0.00 | 1.04E-02 | 9.65E-01 |
| VRr | audSpec_Rfilt_sma[20] | 0.00 | 1.05E-02 | 8.03E-01 |
| VRr | audSpec_Rfilt_sma[21] | 0.01 | 1.07E-02 | 4.11E-01 |
| VRr | audSpec_Rfilt_sma[22] | 0.00 | 1.03E-02 | 9.46E-01 |
| VRr | audSpec_Rfilt_sma[23] | -0.01 | 9.99E-03 | 3.31E-01 |
| VRr | audSpec_Rfilt_sma[24] | -0.01 | 9.78E-03 | 1.90E-01 |
| VRr | audSpec_Rfilt_sma[25] | -0.01 | 9.79E-03 | 3.95E-01 |
| VRr | pcm_fftMag_fband250-650_sma | 0.00 | 9.79E-03 | 6.34E-01 |
| VRr | pcm_fftMag_fband1000-4000_sma | -0.02 | 9.86E-03 | 1.71E-02 |
| VRr | pcm_fftMag_spectralRollOff25.0_sma | -0.03 | 9.57E-03 | 2.66E-04 |
| VRr | pcm_fftMag_spectralRollOff50.0_sma | -0.04 | 9.55E-03 | 2.14E-04 |
| VRr | pcm_fftMag_spectralRollOff75.0_sma | -0.04 | 9.55E-03 | 1.74E-04 |
| VRr | pcm_fftMag_spectralRollOff90.0_sma | -0.04 | 9.58E-03 | 2.55E-04 |
| VRr | pcm_fftMag_spectralFlux_sma | -0.01 | 9.84E-03 | 1.29E-01 |
| VRr | pcm_fftMag_spectralCentroid_sma | -0.04 | 9.55E-03 | 2.20E-04 |
| VRr | pcm_fftMag_spectralEntropy_sma | -0.03 | 9.61E-03 | 3.43E-04 |
| VRr | pcm_fftMag_spectralVariance_sma | -0.03 | 9.52E-03 | 1.03E-03 |
| VRr | pcm_fftMag_spectralSkewness_sma | 0.03 | 9.39E-03 | 5.11E-04 |
| VRr | pcm_fftMag_spectralKurtosis_sma | 0.03 | 1.01E-02 | 1.25E-02 |
| VRr | pcm_fftMag_spectralSlope_sma | 0.01 | 1.07E-02 | 3.54E-01 |
| VRr | pcm_fftMag_psySharpness_sma | -0.04 | 9.58E-03 | 2.09E-04 |
| VRr | pcm_fftMag_spectralHarmonicity_sma | -0.01 | 9.77E-03 | 1.69E-01 |
| VRr | mfcc_sma[1] | 0.03 | 9.56E-03 | 7.40E-03 |
| VRr | mfcc_sma[2] | 0.03 | 9.46E-03 | 4.19E-03 |
| VRr | mfcc_sma[3] | 0.02 | 1.01E-02 | 7.76E-02 |
| VRr | mfcc_sma[4] | 0.03 | 9.69E-03 | 6.60E-04 |
| VRr | mfcc_sma[5] | -0.02 | 9.69E-03 | 1.08E-01 |
| VRr | mfcc_sma[6] | 0.03 | 9.69E-03 | 6.09E-03 |
| VRr | mfcc_sma[7] | 0.00 | 9.34E-03 | 6.04E-01 |
| VRr | mfcc_sma[8] | 0.03 | 9.69E-03 | 6.94E-03 |
| VRr | mfcc_sma[9] | 0.01 | 9.86E-03 | 1.40E-01 |
| VRr | mfcc_sma[10] | 0.00 | 9.37E-03 | 7.87E-01 |
| VRr | mfcc_sma[11] | 0.03 | 9.50E-03 | 6.15E-03 |
| VRr | mfcc_sma[12] | 0.00 | 9.42E-03 | 7.14E-01 |
| VRr | mfcc_sma[13] | 0.02 | 9.70E-03 | 2.76E-02 |
| VRr | mfcc_sma[14] | 0.01 | 9.84E-03 | 4.09E-01 |
|  |  |  |  |  |
| **PASi** | F0final_sma | -0.03 | 9.63E-03 | 1.08E-03 |
| PASi | voicingFinalUnclipped_sma | 0.01 | 8.67E-03 | 3.22E-01 |
| PASi | jitterLocal_sma | -0.01 | 8.91E-03 | 3.69E-01 |
| PASi | jitterDDP_sma | -0.01 | 8.75E-03 | 4.30E-01 |
| PASi | shimmerLocal_sma | -0.01 | 9.21E-03 | 1.77E-01 |
| PASi | logHNR_sma | -0.01 | 9.29E-03 | 1.58E-01 |
| PASi | audspec_lengthL1norm_sma | -0.01 | 8.86E-03 | 3.10E-01 |
| PASi | audspecRasta_lengthL1norm_sma | -0.01 | 9.74E-03 | 2.25E-01 |
| PASi | pcm_RMSenergy_sma | -0.01 | 8.80E-03 | 9.64E-02 |
| PASi | pcm_zcr_sma | 0.00 | 8.36E-03 | 5.98E-01 |
| PASi | audSpec_Rfilt_sma[0] | -0.02 | 9.21E-03 | 7.94E-02 |
| PASi | audSpec_Rfilt_sma[1] | -0.03 | 9.33E-03 | 4.69E-03 |
| PASi | audSpec_Rfilt_sma[2] | -0.02 | 9.55E-03 | 3.43E-02 |
| PASi | audSpec_Rfilt_sma[3] | -0.02 | 9.66E-03 | 3.86E-02 |
| PASi | audSpec_Rfilt_sma[4] | -0.02 | 9.58E-03 | 5.11E-02 |
| PASi | audSpec_Rfilt_sma[5] | -0.02 | 9.45E-03 | 1.97E-02 |
| PASi | audSpec_Rfilt_sma[6] | -0.02 | 9.41E-03 | 2.01E-02 |
| PASi | audSpec_Rfilt_sma[7] | -0.02 | 9.30E-03 | 2.88E-02 |
| PASi | audSpec_Rfilt_sma[8] | -0.02 | 9.19E-03 | 2.03E-02 |
| PASi | audSpec_Rfilt_sma[9] | -0.02 | 9.22E-03 | 2.20E-02 |
| PASi | audSpec_Rfilt_sma[10] | -0.02 | 9.24E-03 | 2.60E-02 |
| PASi | audSpec_Rfilt_sma[11] | -0.02 | 9.36E-03 | 4.58E-02 |
| PASi | audSpec_Rfilt_sma[12] | -0.01 | 9.47E-03 | 1.57E-01 |
| PASi | audSpec_Rfilt_sma[13] | -0.01 | 9.64E-03 | 1.51E-01 |
| PASi | audSpec_Rfilt_sma[14] | -0.02 | 9.62E-03 | 6.73E-02 |
| PASi | audSpec_Rfilt_sma[15] | -0.01 | 9.59E-03 | 2.32E-01 |
| PASi | audSpec_Rfilt_sma[16] | 0.00 | 9.52E-03 | 8.07E-01 |
| PASi | audSpec_Rfilt_sma[17] | 0.00 | 9.49E-03 | 6.24E-01 |
| PASi | audSpec_Rfilt_sma[18] | 0.00 | 9.43E-03 | 9.51E-01 |
| PASi | audSpec_Rfilt_sma[19] | 0.01 | 9.26E-03 | 4.32E-01 |
| PASi | audSpec_Rfilt_sma[20] | 0.00 | 9.36E-03 | 6.30E-01 |
| PASi | audSpec_Rfilt_sma[21] | 0.00 | 9.57E-03 | 7.85E-01 |
| PASi | audSpec_Rfilt_sma[22] | 0.00 | 9.28E-03 | 6.88E-01 |
| PASi | audSpec_Rfilt_sma[23] | 0.01 | 9.00E-03 | 4.11E-01 |
| PASi | audSpec_Rfilt_sma[24] | 0.01 | 8.84E-03 | 2.71E-01 |
| PASi | audSpec_Rfilt_sma[25] | 0.01 | 8.83E-03 | 3.09E-01 |
| PASi | pcm_fftMag_fband250-650_sma | -0.02 | 8.76E-03 | 4.97E-03 |
| PASi | pcm_fftMag_fband1000-4000_sma | -0.02 | 8.95E-03 | 5.60E-02 |
| PASi | pcm_fftMag_spectralRollOff25.0_sma | 0.00 | 8.46E-03 | 9.36E-01 |
| PASi | pcm_fftMag_spectralRollOff50.0_sma | 0.00 | 8.43E-03 | 8.97E-01 |
| PASi | pcm_fftMag_spectralRollOff75.0_sma | 0.00 | 8.42E-03 | 7.04E-01 |
| PASi | pcm_fftMag_spectralRollOff90.0_sma | 0.00 | 8.45E-03 | 5.91E-01 |
| PASi | pcm_fftMag_spectralFlux_sma | -0.01 | 8.85E-03 | 1.65E-01 |
| PASi | pcm_fftMag_spectralCentroid_sma | 0.00 | 8.41E-03 | 7.27E-01 |
| PASi | pcm_fftMag_spectralEntropy_sma | 0.00 | 8.46E-03 | 8.41E-01 |
| PASi | pcm_fftMag_spectralVariance_sma | 0.01 | 8.37E-03 | 3.46E-01 |
| PASi | pcm_fftMag_spectralSkewness_sma | 0.01 | 8.18E-03 | 3.90E-01 |
| PASi | pcm_fftMag_spectralKurtosis_sma | 0.01 | 8.16E-03 | 1.58E-01 |
| PASi | pcm_fftMag_spectralSlope_sma | 0.01 | 8.64E-03 | 1.60E-01 |
| PASi | pcm_fftMag_psySharpness_sma | 0.00 | 8.44E-03 | 8.51E-01 |
| PASi | pcm_fftMag_spectralHarmonicity_sma | -0.03 | 8.79E-03 | 2.43E-03 |
| PASi | mfcc_sma[1] | -0.01 | 8.41E-03 | 1.10E-01 |
| PASi | mfcc_sma[2] | 0.02 | 8.50E-03 | 6.26E-02 |
| PASi | mfcc_sma[3] | -0.01 | 9.00E-03 | 3.17E-01 |
| PASi | mfcc_sma[4] | 0.00 | 8.67E-03 | 7.75E-01 |
| PASi | mfcc_sma[5] | 0.02 | 8.69E-03 | 1.72E-02 |
| PASi | mfcc_sma[6] | -0.01 | 8.60E-03 | 1.90E-01 |
| PASi | mfcc_sma[7] | 0.03 | 8.25E-03 | 8.00E-04 |
| PASi | mfcc_sma[8] | 0.00 | 8.68E-03 | 7.81E-01 |
| PASi | mfcc_sma[9] | 0.01 | 8.99E-03 | 4.59E-01 |
| PASi | mfcc_sma[10] | 0.03 | 8.30E-03 | 1.12E-03 |
| PASi | mfcc_sma[11] | 0.00 | 8.41E-03 | 6.90E-01 |
| PASi | mfcc_sma[12] | 0.02 | 8.49E-03 | 4.12E-02 |
| PASi | mfcc_sma[13] | 0.01 | 8.88E-03 | 2.84E-01 |
| PASi | mfcc_sma[14] | -0.02 | 8.86E-03 | 2.00E-02 |
|  |  |  |  |  |
| **PASd** | F0final_sma | -0.03 | 9.96E-03 | 9.88E-03 |
| PASd | voicingFinalUnclipped_sma | -0.01 | 8.91E-03 | 1.58E-01 |
| PASd | jitterLocal_sma | -0.01 | 9.30E-03 | 2.82E-01 |
| PASd | jitterDDP_sma | -0.01 | 9.15E-03 | 2.71E-01 |
| PASd | shimmerLocal_sma | -0.02 | 9.54E-03 | 9.80E-02 |
| PASd | logHNR_sma | -0.02 | 9.61E-03 | 4.78E-02 |
| PASd | audspec_lengthL1norm_sma | -0.01 | 9.15E-03 | 1.43E-01 |
| PASd | audspecRasta_lengthL1norm_sma | -0.01 | 1.01E-02 | 4.19E-01 |
| PASd | pcm_RMSenergy_sma | -0.02 | 9.08E-03 | 7.53E-02 |
| PASd | pcm_zcr_sma | 0.00 | 8.71E-03 | 8.62E-01 |
| PASd | audSpec_Rfilt_sma[0] | -0.02 | 9.51E-03 | 4.19E-02 |
| PASd | audSpec_Rfilt_sma[1] | -0.03 | 9.68E-03 | 2.15E-03 |
| PASd | audSpec_Rfilt_sma[2] | -0.01 | 9.92E-03 | 1.48E-01 |
| PASd | audSpec_Rfilt_sma[3] | -0.01 | 1.00E-02 | 2.88E-01 |
| PASd | audSpec_Rfilt_sma[4] | -0.01 | 9.92E-03 | 2.38E-01 |
| PASd | audSpec_Rfilt_sma[5] | -0.01 | 9.81E-03 | 2.00E-01 |
| PASd | audSpec_Rfilt_sma[6] | -0.01 | 9.78E-03 | 2.19E-01 |
| PASd | audSpec_Rfilt_sma[7] | -0.01 | 9.67E-03 | 3.85E-01 |
| PASd | audSpec_Rfilt_sma[8] | -0.01 | 9.55E-03 | 4.01E-01 |
| PASd | audSpec_Rfilt_sma[9] | -0.01 | 9.57E-03 | 4.28E-01 |
| PASd | audSpec_Rfilt_sma[10] | -0.01 | 9.59E-03 | 3.66E-01 |
| PASd | audSpec_Rfilt_sma[11] | -0.01 | 9.71E-03 | 3.50E-01 |
| PASd | audSpec_Rfilt_sma[12] | -0.01 | 9.82E-03 | 3.76E-01 |
| PASd | audSpec_Rfilt_sma[13] | -0.01 | 9.99E-03 | 2.63E-01 |
| PASd | audSpec_Rfilt_sma[14] | -0.01 | 9.99E-03 | 1.52E-01 |
| PASd | audSpec_Rfilt_sma[15] | -0.01 | 9.94E-03 | 3.98E-01 |
| PASd | audSpec_Rfilt_sma[16] | 0.00 | 9.88E-03 | 8.37E-01 |
| PASd | audSpec_Rfilt_sma[17] | -0.01 | 9.87E-03 | 4.92E-01 |
| PASd | audSpec_Rfilt_sma[18] | 0.00 | 9.81E-03 | 8.46E-01 |
| PASd | audSpec_Rfilt_sma[19] | 0.00 | 9.64E-03 | 7.86E-01 |
| PASd | audSpec_Rfilt_sma[20] | 0.00 | 9.71E-03 | 9.00E-01 |
| PASd | audSpec_Rfilt_sma[21] | 0.00 | 9.90E-03 | 8.67E-01 |
| PASd | audSpec_Rfilt_sma[22] | 0.00 | 9.61E-03 | 8.51E-01 |
| PASd | audSpec_Rfilt_sma[23] | 0.00 | 9.31E-03 | 8.97E-01 |
| PASd | audSpec_Rfilt_sma[24] | 0.00 | 9.12E-03 | 9.96E-01 |
| PASd | audSpec_Rfilt_sma[25] | 0.00 | 9.11E-03 | 8.21E-01 |
| PASd | pcm_fftMag_fband250-650_sma | -0.02 | 9.05E-03 | 4.08E-02 |
| PASd | pcm_fftMag_fband1000-4000_sma | -0.02 | 9.22E-03 | 6.73E-02 |
| PASd | pcm_fftMag_spectralRollOff25.0_sma | 0.00 | 8.79E-03 | 6.06E-01 |
| PASd | pcm_fftMag_spectralRollOff50.0_sma | 0.00 | 8.76E-03 | 7.60E-01 |
| PASd | pcm_fftMag_spectralRollOff75.0_sma | 0.00 | 8.76E-03 | 8.27E-01 |
| PASd | pcm_fftMag_spectralRollOff90.0_sma | 0.00 | 8.79E-03 | 7.92E-01 |
| PASd | pcm_fftMag_spectralFlux_sma | -0.01 | 9.12E-03 | 1.14E-01 |
| PASd | pcm_fftMag_spectralCentroid_sma | 0.00 | 8.75E-03 | 7.77E-01 |
| PASd | pcm_fftMag_spectralEntropy_sma | 0.00 | 8.80E-03 | 6.75E-01 |
| PASd | pcm_fftMag_spectralVariance_sma | 0.00 | 8.72E-03 | 9.59E-01 |
| PASd | pcm_fftMag_spectralSkewness_sma | 0.01 | 8.52E-03 | 2.46E-01 |
| PASd | pcm_fftMag_spectralKurtosis_sma | 0.01 | 8.52E-03 | 1.63E-01 |
| PASd | pcm_fftMag_spectralSlope_sma | 0.01 | 8.95E-03 | 2.00E-01 |
| PASd | pcm_fftMag_psySharpness_sma | 0.00 | 8.78E-03 | 6.77E-01 |
| PASd | pcm_fftMag_spectralHarmonicity_sma | -0.02 | 9.07E-03 | 1.27E-02 |
| PASd | mfcc_sma[1] | -0.01 | 8.77E-03 | 3.74E-01 |
| PASd | mfcc_sma[2] | 0.01 | 8.76E-03 | 9.01E-02 |
| PASd | mfcc_sma[3] | 0.00 | 9.34E-03 | 6.87E-01 |
| PASd | mfcc_sma[4] | 0.00 | 8.97E-03 | 9.74E-01 |
| PASd | mfcc_sma[5] | 0.01 | 8.99E-03 | 1.19E-01 |
| PASd | mfcc_sma[6] | -0.01 | 8.94E-03 | 3.74E-01 |
| PASd | mfcc_sma[7] | 0.02 | 8.59E-03 | 2.99E-02 |
| PASd | mfcc_sma[8] | 0.00 | 8.97E-03 | 9.34E-01 |
| PASd | mfcc_sma[9] | 0.00 | 9.21E-03 | 6.07E-01 |
| PASd | mfcc_sma[10] | 0.02 | 8.64E-03 | 1.52E-02 |
| PASd | mfcc_sma[11] | 0.00 | 8.73E-03 | 9.50E-01 |
| PASd | mfcc_sma[12] | 0.01 | 8.74E-03 | 1.33E-01 |
| PASd | mfcc_sma[13] | 0.01 | 9.09E-03 | 1.04E-01 |
| PASd | mfcc_sma[14] | 0.00 | 9.16E-03 | 7.62E-01 |
|  |  |  |  |  |
| **PASr** | F0final_sma | -0.03 | 1.15E-02 | 6.90E-03 |
| PASr | voicingFinalUnclipped_sma | 0.01 | 1.04E-02 | 4.28E-01 |
| PASr | jitterLocal_sma | 0.00 | 1.09E-02 | 7.11E-01 |
| PASr | jitterDDP_sma | 0.00 | 1.07E-02 | 7.75E-01 |
| PASr | shimmerLocal_sma | -0.01 | 1.11E-02 | 3.31E-01 |
| PASr | logHNR_sma | -0.02 | 1.11E-02 | 4.85E-02 |
| PASr | audspec_lengthL1norm_sma | -0.02 | 1.06E-02 | 4.69E-02 |
| PASr | audspecRasta_lengthL1norm_sma | -0.01 | 1.17E-02 | 4.94E-01 |
| PASr | pcm_RMSenergy_sma | -0.02 | 1.05E-02 | 1.16E-01 |
| PASr | pcm_zcr_sma | -0.03 | 1.04E-02 | 8.68E-04 |
| PASr | audSpec_Rfilt_sma[0] | -0.04 | 1.09E-02 | 1.61E-04 |
| PASr | audSpec_Rfilt_sma[1] | -0.07 | 1.12E-02 | 2.26E-10 |
| PASr | audSpec_Rfilt_sma[2] | -0.06 | 1.16E-02 | 1.23E-06 |
| PASr | audSpec_Rfilt_sma[3] | -0.02 | 1.17E-02 | 3.83E-02 |
| PASr | audSpec_Rfilt_sma[4] | -0.01 | 1.16E-02 | 5.60E-01 |
| PASr | audSpec_Rfilt_sma[5] | 0.00 | 1.15E-02 | 8.27E-01 |
| PASr | audSpec_Rfilt_sma[6] | 0.01 | 1.15E-02 | 5.27E-01 |
| PASr | audSpec_Rfilt_sma[7] | 0.01 | 1.14E-02 | 3.45E-01 |
| PASr | audSpec_Rfilt_sma[8] | 0.02 | 1.13E-02 | 1.80E-01 |
| PASr | audSpec_Rfilt_sma[9] | 0.02 | 1.13E-02 | 1.45E-01 |
| PASr | audSpec_Rfilt_sma[10] | 0.01 | 1.13E-02 | 2.11E-01 |
| PASr | audSpec_Rfilt_sma[11] | 0.01 | 1.14E-02 | 4.82E-01 |
| PASr | audSpec_Rfilt_sma[12] | 0.00 | 1.15E-02 | 8.82E-01 |
| PASr | audSpec_Rfilt_sma[13] | 0.00 | 1.17E-02 | 7.83E-01 |
| PASr | audSpec_Rfilt_sma[14] | 0.00 | 1.16E-02 | 7.37E-01 |
| PASr | audSpec_Rfilt_sma[15] | 0.00 | 1.16E-02 | 7.24E-01 |
| PASr | audSpec_Rfilt_sma[16] | -0.01 | 1.15E-02 | 3.05E-01 |
| PASr | audSpec_Rfilt_sma[17] | -0.02 | 1.14E-02 | 1.68E-01 |
| PASr | audSpec_Rfilt_sma[18] | -0.02 | 1.14E-02 | 1.77E-01 |
| PASr | audSpec_Rfilt_sma[19] | -0.02 | 1.12E-02 | 1.47E-01 |
| PASr | audSpec_Rfilt_sma[20] | -0.02 | 1.13E-02 | 7.31E-02 |
| PASr | audSpec_Rfilt_sma[21] | -0.01 | 1.15E-02 | 2.28E-01 |
| PASr | audSpec_Rfilt_sma[22] | -0.02 | 1.11E-02 | 1.25E-01 |
| PASr | audSpec_Rfilt_sma[23] | -0.02 | 1.07E-02 | 3.04E-02 |
| PASr | audSpec_Rfilt_sma[24] | -0.02 | 1.05E-02 | 2.21E-02 |
| PASr | audSpec_Rfilt_sma[25] | -0.02 | 1.05E-02 | 3.49E-02 |
| PASr | pcm_fftMag_fband250-650_sma | -0.02 | 1.05E-02 | 1.44E-01 |
| PASr | pcm_fftMag_fband1000-4000_sma | -0.03 | 1.06E-02 | 9.51E-03 |
| PASr | pcm_fftMag_spectralRollOff25.0_sma | -0.03 | 1.04E-02 | 1.08E-03 |
| PASr | pcm_fftMag_spectralRollOff50.0_sma | -0.04 | 1.04E-02 | 3.51E-04 |
| PASr | pcm_fftMag_spectralRollOff75.0_sma | -0.04 | 1.04E-02 | 3.32E-04 |
| PASr | pcm_fftMag_spectralRollOff90.0_sma | -0.03 | 1.04E-02 | 9.54E-04 |
| PASr | pcm_fftMag_spectralFlux_sma | -0.02 | 1.05E-02 | 1.33E-01 |
| PASr | pcm_fftMag_spectralCentroid_sma | -0.04 | 1.04E-02 | 6.66E-04 |
| PASr | pcm_fftMag_spectralEntropy_sma | -0.03 | 1.05E-02 | 2.80E-03 |
| PASr | pcm_fftMag_spectralVariance_sma | -0.03 | 1.03E-02 | 2.53E-03 |
| PASr | pcm_fftMag_spectralSkewness_sma | 0.02 | 1.01E-02 | 1.61E-02 |
| PASr | pcm_fftMag_spectralKurtosis_sma | 0.02 | 1.01E-02 | 9.93E-02 |
| PASr | pcm_fftMag_spectralSlope_sma | 0.02 | 1.04E-02 | 1.27E-01 |
| PASr | pcm_fftMag_psySharpness_sma | -0.03 | 1.04E-02 | 1.08E-03 |
| PASr | pcm_fftMag_spectralHarmonicity_sma | -0.03 | 1.05E-02 | 1.59E-02 |
| PASr | mfcc_sma[1] | 0.03 | 1.04E-02 | 1.13E-02 |
| PASr | mfcc_sma[2] | 0.02 | 1.03E-02 | 2.57E-02 |
| PASr | mfcc_sma[3] | 0.02 | 1.09E-02 | 1.01E-01 |
| PASr | mfcc_sma[4] | 0.02 | 1.05E-02 | 6.42E-02 |
| PASr | mfcc_sma[5] | -0.01 | 1.05E-02 | 3.02E-01 |
| PASr | mfcc_sma[6] | 0.03 | 1.05E-02 | 1.29E-02 |
| PASr | mfcc_sma[7] | -0.02 | 1.01E-02 | 1.13E-01 |
| PASr | mfcc_sma[8] | 0.04 | 1.05E-02 | 2.86E-04 |
| PASr | mfcc_sma[9] | -0.01 | 1.06E-02 | 4.60E-01 |
| PASr | mfcc_sma[10] | -0.02 | 1.01E-02 | 1.30E-01 |
| PASr | mfcc_sma[11] | 0.03 | 1.03E-02 | 2.91E-03 |
| PASr | mfcc_sma[12] | -0.02 | 1.02E-02 | 4.89E-02 |
| PASr | mfcc_sma[13] | 0.02 | 1.05E-02 | 7.24E-02 |
| PASr | mfcc_sma[14] | 0.02 | 1.06E-02 | 1.33E-01 |
|  |  |  |  |  |
| **DSF** | F0final_sma | 0.04 | 1.09E-02 | 1.59E-04 |
| DSF | voicingFinalUnclipped_sma | 0.03 | 9.72E-03 | 1.99E-03 |
| DSF | jitterLocal_sma | 0.03 | 1.04E-02 | 7.48E-04 |
| DSF | jitterDDP_sma | 0.03 | 1.03E-02 | 2.38E-03 |
| DSF | shimmerLocal_sma | 0.04 | 1.05E-02 | 4.01E-04 |
| DSF | logHNR_sma | 0.03 | 1.06E-02 | 7.69E-03 |
| DSF | audspec_lengthL1norm_sma | 0.00 | 9.98E-03 | 8.30E-01 |
| DSF | audspecRasta_lengthL1norm_sma | 0.06 | 1.10E-02 | 1.16E-08 |
| DSF | pcm_RMSenergy_sma | 0.04 | 9.90E-03 | 3.63E-04 |
| DSF | pcm_zcr_sma | -0.05 | 9.53E-03 | 3.57E-08 |
| DSF | audSpec_Rfilt_sma[0] | 0.06 | 1.06E-02 | 5.65E-09 |
| DSF | audSpec_Rfilt_sma[1] | 0.05 | 1.07E-02 | 1.17E-05 |
| DSF | audSpec_Rfilt_sma[2] | 0.05 | 1.10E-02 | 6.92E-06 |
| DSF | audSpec_Rfilt_sma[3] | 0.07 | 1.11E-02 | 2.93E-09 |
| DSF | audSpec_Rfilt_sma[4] | 0.07 | 1.10E-02 | 2.57E-11 |
| DSF | audSpec_Rfilt_sma[5] | 0.09 | 1.08E-02 | 2.74E-15 |
| DSF | audSpec_Rfilt_sma[6] | 0.09 | 1.07E-02 | 4.81E-17 |
| DSF | audSpec_Rfilt_sma[7] | 0.08 | 1.06E-02 | 6.90E-13 |
| DSF | audSpec_Rfilt_sma[8] | 0.07 | 1.04E-02 | 8.58E-11 |
| DSF | audSpec_Rfilt_sma[9] | 0.07 | 1.05E-02 | 1.43E-12 |
| DSF | audSpec_Rfilt_sma[10] | 0.08 | 1.05E-02 | 1.33E-13 |
| DSF | audSpec_Rfilt_sma[11] | 0.07 | 1.06E-02 | 2.68E-11 |
| DSF | audSpec_Rfilt_sma[12] | 0.05 | 1.07E-02 | 4.82E-07 |
| DSF | audSpec_Rfilt_sma[13] | 0.05 | 1.09E-02 | 6.53E-06 |
| DSF | audSpec_Rfilt_sma[14] | 0.05 | 1.09E-02 | 2.60E-06 |
| DSF | audSpec_Rfilt_sma[15] | 0.04 | 1.08E-02 | 4.91E-05 |
| DSF | audSpec_Rfilt_sma[16] | 0.02 | 1.08E-02 | 4.52E-02 |
| DSF | audSpec_Rfilt_sma[17] | 0.02 | 1.07E-02 | 1.54E-01 |
| DSF | audSpec_Rfilt_sma[18] | 0.01 | 1.07E-02 | 2.03E-01 |
| DSF | audSpec_Rfilt_sma[19] | 0.02 | 1.05E-02 | 1.51E-01 |
| DSF | audSpec_Rfilt_sma[20] | 0.04 | 1.06E-02 | 8.78E-04 |
| DSF | audSpec_Rfilt_sma[21] | 0.07 | 1.08E-02 | 4.94E-10 |
| DSF | audSpec_Rfilt_sma[22] | 0.05 | 1.06E-02 | 1.69E-05 |
| DSF | audSpec_Rfilt_sma[23] | 0.02 | 1.02E-02 | 3.68E-02 |
| DSF | audSpec_Rfilt_sma[24] | 0.00 | 9.95E-03 | 6.26E-01 |
| DSF | audSpec_Rfilt_sma[25] | 0.02 | 9.96E-03 | 2.15E-02 |
| DSF | pcm_fftMag_fband250-650_sma | 0.07 | 9.87E-03 | 3.45E-13 |
| DSF | pcm_fftMag_fband1000-4000_sma | 0.01 | 1.00E-02 | 2.99E-01 |
| DSF | pcm_fftMag_spectralRollOff25.0_sma | -0.04 | 9.61E-03 | 1.26E-04 |
| DSF | pcm_fftMag_spectralRollOff50.0_sma | -0.04 | 9.59E-03 | 2.70E-06 |
| DSF | pcm_fftMag_spectralRollOff75.0_sma | -0.05 | 9.58E-03 | 2.88E-07 |
| DSF | pcm_fftMag_spectralRollOff90.0_sma | -0.05 | 9.62E-03 | 1.01E-07 |
| DSF | pcm_fftMag_spectralFlux_sma | 0.03 | 9.95E-03 | 1.04E-02 |
| DSF | pcm_fftMag_spectralCentroid_sma | -0.05 | 9.58E-03 | 4.32E-07 |
| DSF | pcm_fftMag_spectralEntropy_sma | -0.05 | 9.63E-03 | 1.09E-07 |
| DSF | pcm_fftMag_spectralVariance_sma | -0.05 | 9.57E-03 | 2.73E-08 |
| DSF | pcm_fftMag_spectralSkewness_sma | 0.03 | 9.34E-03 | 3.02E-03 |
| DSF | pcm_fftMag_spectralKurtosis_sma | 0.01 | 9.38E-03 | 3.74E-01 |
| DSF | pcm_fftMag_spectralSlope_sma | -0.05 | 9.77E-03 | 1.70E-07 |
| DSF | pcm_fftMag_psySharpness_sma | -0.05 | 9.61E-03 | 9.33E-07 |
| DSF | pcm_fftMag_spectralHarmonicity_sma | 0.06 | 9.87E-03 | 4.41E-09 |
| DSF | mfcc_sma[1] | 0.06 | 9.66E-03 | 6.41E-11 |
| DSF | mfcc_sma[2] | 0.02 | 9.60E-03 | 1.16E-01 |
| DSF | mfcc_sma[3] | 0.06 | 1.03E-02 | 9.36E-09 |
| DSF | mfcc_sma[4] | 0.03 | 9.78E-03 | 4.04E-03 |
| DSF | mfcc_sma[5] | -0.05 | 9.87E-03 | 2.17E-07 |
| DSF | mfcc_sma[6] | 0.05 | 9.83E-03 | 8.75E-08 |
| DSF | mfcc_sma[7] | -0.07 | 9.48E-03 | 2.23E-12 |
| DSF | mfcc_sma[8] | 0.05 | 9.85E-03 | 3.53E-08 |
| DSF | mfcc_sma[9] | -0.02 | 1.01E-02 | 6.97E-02 |
| DSF | mfcc_sma[10] | -0.06 | 9.52E-03 | 1.65E-09 |
| DSF | mfcc_sma[11] | 0.05 | 9.64E-03 | 8.80E-08 |
| DSF | mfcc_sma[12] | -0.06 | 9.62E-03 | 1.69E-09 |
| DSF | mfcc_sma[13] | 0.00 | 9.94E-03 | 6.94E-01 |
| DSF | mfcc_sma[14] | 0.02 | 1.01E-02 | 2.32E-02 |
|  |  |  |  |  |
| **DSB** | F0final_sma | 0.01 | 1.11E-02 | 2.62E-01 |
| DSB | voicingFinalUnclipped_sma | -0.01 | 9.81E-03 | 5.04E-01 |
| DSB | jitterLocal_sma | 0.01 | 1.04E-02 | 1.59E-01 |
| DSB | jitterDDP_sma | 0.01 | 1.03E-02 | 2.25E-01 |
| DSB | shimmerLocal_sma | 0.02 | 1.06E-02 | 1.34E-01 |
| DSB | logHNR_sma | 0.01 | 1.07E-02 | 2.75E-01 |
| DSB | audspec_lengthL1norm_sma | 0.00 | 1.01E-02 | 7.61E-01 |
| DSB | audspecRasta_lengthL1norm_sma | 0.05 | 1.12E-02 | 1.94E-05 |
| DSB | pcm_RMSenergy_sma | 0.02 | 1.00E-02 | 6.86E-02 |
| DSB | pcm_zcr_sma | -0.04 | 9.69E-03 | 2.08E-05 |
| DSB | audSpec_Rfilt_sma[0] | 0.03 | 1.06E-02 | 1.79E-02 |
| DSB | audSpec_Rfilt_sma[1] | 0.02 | 1.08E-02 | 3.54E-02 |
| DSB | audSpec_Rfilt_sma[2] | 0.05 | 1.10E-02 | 2.37E-05 |
| DSB | audSpec_Rfilt_sma[3] | 0.05 | 1.11E-02 | 1.66E-06 |
| DSB | audSpec_Rfilt_sma[4] | 0.06 | 1.11E-02 | 1.65E-07 |
| DSB | audSpec_Rfilt_sma[5] | 0.06 | 1.10E-02 | 1.20E-08 |
| DSB | audSpec_Rfilt_sma[6] | 0.06 | 1.09E-02 | 1.67E-08 |
| DSB | audSpec_Rfilt_sma[7] | 0.05 | 1.07E-02 | 3.09E-07 |
| DSB | audSpec_Rfilt_sma[8] | 0.05 | 1.06E-02 | 1.36E-06 |
| DSB | audSpec_Rfilt_sma[9] | 0.06 | 1.06E-02 | 1.84E-07 |
| DSB | audSpec_Rfilt_sma[10] | 0.06 | 1.06E-02 | 4.34E-08 |
| DSB | audSpec_Rfilt_sma[11] | 0.05 | 1.08E-02 | 4.44E-07 |
| DSB | audSpec_Rfilt_sma[12] | 0.05 | 1.09E-02 | 1.08E-05 |
| DSB | audSpec_Rfilt_sma[13] | 0.04 | 1.11E-02 | 1.50E-04 |
| DSB | audSpec_Rfilt_sma[14] | 0.04 | 1.11E-02 | 3.26E-04 |
| DSB | audSpec_Rfilt_sma[15] | 0.04 | 1.10E-02 | 9.70E-04 |
| DSB | audSpec_Rfilt_sma[16] | 0.03 | 1.09E-02 | 1.97E-02 |
| DSB | audSpec_Rfilt_sma[17] | 0.02 | 1.09E-02 | 4.99E-02 |
| DSB | audSpec_Rfilt_sma[18] | 0.02 | 1.09E-02 | 6.22E-02 |
| DSB | audSpec_Rfilt_sma[19] | 0.02 | 1.07E-02 | 3.74E-02 |
| DSB | audSpec_Rfilt_sma[20] | 0.03 | 1.08E-02 | 1.54E-02 |
| DSB | audSpec_Rfilt_sma[21] | 0.04 | 1.10E-02 | 4.33E-04 |
| DSB | audSpec_Rfilt_sma[22] | 0.02 | 1.07E-02 | 2.98E-02 |
| DSB | audSpec_Rfilt_sma[23] | 0.01 | 1.03E-02 | 5.48E-01 |
| DSB | audSpec_Rfilt_sma[24] | 0.00 | 1.01E-02 | 7.82E-01 |
| DSB | audSpec_Rfilt_sma[25] | 0.01 | 1.01E-02 | 4.41E-01 |
| DSB | pcm_fftMag_fband250-650_sma | 0.03 | 1.00E-02 | 5.94E-04 |
| DSB | pcm_fftMag_fband1000-4000_sma | 0.00 | 1.02E-02 | 6.41E-01 |
| DSB | pcm_fftMag_spectralRollOff25.0_sma | -0.04 | 9.75E-03 | 1.93E-04 |
| DSB | pcm_fftMag_spectralRollOff50.0_sma | -0.04 | 9.72E-03 | 3.66E-05 |
| DSB | pcm_fftMag_spectralRollOff75.0_sma | -0.04 | 9.73E-03 | 2.97E-05 |
| DSB | pcm_fftMag_spectralRollOff90.0_sma | -0.04 | 9.77E-03 | 3.67E-05 |
| DSB | pcm_fftMag_spectralFlux_sma | 0.01 | 1.00E-02 | 1.96E-01 |
| DSB | pcm_fftMag_spectralCentroid_sma | -0.04 | 9.72E-03 | 3.90E-05 |
| DSB | pcm_fftMag_spectralEntropy_sma | -0.04 | 9.77E-03 | 1.11E-05 |
| DSB | pcm_fftMag_spectralVariance_sma | -0.04 | 9.72E-03 | 1.84E-04 |
| DSB | pcm_fftMag_spectralSkewness_sma | 0.03 | 9.45E-03 | 5.99E-04 |
| DSB | pcm_fftMag_spectralKurtosis_sma | 0.02 | 9.46E-03 | 4.06E-02 |
| DSB | pcm_fftMag_spectralSlope_sma | -0.03 | 9.90E-03 | 1.34E-03 |
| DSB | pcm_fftMag_psySharpness_sma | -0.04 | 9.75E-03 | 4.03E-05 |
| DSB | pcm_fftMag_spectralHarmonicity_sma | 0.03 | 1.00E-02 | 8.23E-03 |
| DSB | mfcc_sma[1] | 0.03 | 9.85E-03 | 5.79E-04 |
| DSB | mfcc_sma[2] | 0.03 | 9.70E-03 | 8.39E-04 |
| DSB | mfcc_sma[3] | 0.03 | 1.05E-02 | 4.20E-03 |
| DSB | mfcc_sma[4] | 0.03 | 9.92E-03 | 8.03E-03 |
| DSB | mfcc_sma[5] | -0.02 | 1.00E-02 | 2.63E-02 |
| DSB | mfcc_sma[6] | 0.03 | 1.00E-02 | 1.98E-03 |
| DSB | mfcc_sma[7] | -0.03 | 9.64E-03 | 1.81E-03 |
| DSB | mfcc_sma[8] | 0.04 | 9.99E-03 | 8.24E-06 |
| DSB | mfcc_sma[9] | -0.01 | 1.02E-02 | 2.60E-01 |
| DSB | mfcc_sma[10] | -0.03 | 9.68E-03 | 9.52E-03 |
| DSB | mfcc_sma[11] | 0.05 | 9.80E-03 | 6.42E-07 |
| DSB | mfcc_sma[12] | -0.04 | 9.74E-03 | 1.03E-04 |
| DSB | mfcc_sma[13] | 0.03 | 1.00E-02 | 1.50E-03 |
| DSB | mfcc_sma[14] | 0.02 | 1.02E-02 | 4.05E-02 |
|  |  |  |  |  |
| **sim** | F0final_sma | 0.00 | 9.69E-03 | 9.65E-01 |
| sim | voicingFinalUnclipped_sma | 0.02 | 8.53E-03 | 9.20E-03 |
| sim | jitterLocal_sma | 0.01 | 8.95E-03 | 1.29E-01 |
| sim | jitterDDP_sma | 0.01 | 8.80E-03 | 2.51E-01 |
| sim | shimmerLocal_sma | 0.02 | 9.23E-03 | 4.48E-02 |
| sim | logHNR_sma | 0.01 | 9.34E-03 | 2.33E-01 |
| sim | audspec_lengthL1norm_sma | -0.02 | 8.84E-03 | 5.35E-03 |
| sim | audspecRasta_lengthL1norm_sma | 0.01 | 9.75E-03 | 3.05E-01 |
| sim | pcm_RMSenergy_sma | -0.01 | 8.79E-03 | 3.46E-01 |
| sim | pcm_zcr_sma | -0.05 | 8.32E-03 | 1.45E-09 |
| sim | audSpec_Rfilt_sma[0] | -0.01 | 9.17E-03 | 4.25E-01 |
| sim | audSpec_Rfilt_sma[1] | -0.02 | 9.33E-03 | 4.26E-02 |
| sim | audSpec_Rfilt_sma[2] | -0.01 | 9.58E-03 | 2.72E-01 |
| sim | audSpec_Rfilt_sma[3] | 0.01 | 9.66E-03 | 3.84E-01 |
| sim | audSpec_Rfilt_sma[4] | 0.02 | 9.58E-03 | 8.75E-02 |
| sim | audSpec_Rfilt_sma[5] | 0.03 | 9.46E-03 | 2.27E-03 |
| sim | audSpec_Rfilt_sma[6] | 0.03 | 9.43E-03 | 1.10E-03 |
| sim | audSpec_Rfilt_sma[7] | 0.03 | 9.33E-03 | 2.95E-03 |
| sim | audSpec_Rfilt_sma[8] | 0.03 | 9.21E-03 | 1.10E-03 |
| sim | audSpec_Rfilt_sma[9] | 0.03 | 9.23E-03 | 3.47E-04 |
| sim | audSpec_Rfilt_sma[10] | 0.03 | 9.23E-03 | 5.71E-04 |
| sim | audSpec_Rfilt_sma[11] | 0.03 | 9.35E-03 | 1.66E-03 |
| sim | audSpec_Rfilt_sma[12] | 0.03 | 9.47E-03 | 7.37E-03 |
| sim | audSpec_Rfilt_sma[13] | 0.02 | 9.64E-03 | 8.37E-02 |
| sim | audSpec_Rfilt_sma[14] | 0.01 | 9.63E-03 | 3.02E-01 |
| sim | audSpec_Rfilt_sma[15] | 0.00 | 9.58E-03 | 7.34E-01 |
| sim | audSpec_Rfilt_sma[16] | 0.00 | 9.53E-03 | 7.80E-01 |
| sim | audSpec_Rfilt_sma[17] | -0.01 | 9.52E-03 | 5.21E-01 |
| sim | audSpec_Rfilt_sma[18] | -0.01 | 9.44E-03 | 4.49E-01 |
| sim | audSpec_Rfilt_sma[19] | -0.01 | 9.28E-03 | 5.51E-01 |
| sim | audSpec_Rfilt_sma[20] | 0.00 | 9.36E-03 | 6.18E-01 |
| sim | audSpec_Rfilt_sma[21] | 0.01 | 9.56E-03 | 3.98E-01 |
| sim | audSpec_Rfilt_sma[22] | -0.01 | 9.27E-03 | 3.12E-01 |
| sim | audSpec_Rfilt_sma[23] | -0.03 | 8.96E-03 | 3.04E-03 |
| sim | audSpec_Rfilt_sma[24] | -0.03 | 8.77E-03 | 7.13E-05 |
| sim | audSpec_Rfilt_sma[25] | -0.03 | 8.79E-03 | 4.76E-04 |
| sim | pcm_fftMag_fband250-650_sma | 0.00 | 8.74E-03 | 8.62E-01 |
| sim | pcm_fftMag_fband1000-4000_sma | -0.02 | 8.94E-03 | 5.35E-03 |
| sim | pcm_fftMag_spectralRollOff25.0_sma | -0.05 | 8.41E-03 | 7.00E-08 |
| sim | pcm_fftMag_spectralRollOff50.0_sma | -0.05 | 8.37E-03 | 1.39E-09 |
| sim | pcm_fftMag_spectralRollOff75.0_sma | -0.05 | 8.36E-03 | 2.36E-10 |
| sim | pcm_fftMag_spectralRollOff90.0_sma | -0.05 | 8.40E-03 | 4.49E-10 |
| sim | pcm_fftMag_spectralFlux_sma | -0.01 | 8.84E-03 | 2.04E-01 |
| sim | pcm_fftMag_spectralCentroid_sma | -0.05 | 8.36E-03 | 1.19E-09 |
| sim | pcm_fftMag_spectralEntropy_sma | -0.05 | 8.42E-03 | 2.48E-08 |
| sim | pcm_fftMag_spectralVariance_sma | -0.05 | 8.34E-03 | 1.39E-09 |
| sim | pcm_fftMag_spectralSkewness_sma | 0.03 | 8.18E-03 | 3.76E-04 |
| sim | pcm_fftMag_spectralKurtosis_sma | 0.01 | 8.21E-03 | 1.38E-01 |
| sim | pcm_fftMag_spectralSlope_sma | 0.01 | 8.64E-03 | 5.43E-01 |
| sim | pcm_fftMag_psySharpness_sma | -0.05 | 8.40E-03 | 3.91E-09 |
| sim | pcm_fftMag_spectralHarmonicity_sma | -0.01 | 8.77E-03 | 1.61E-01 |
| sim | mfcc_sma[1] | 0.05 | 8.41E-03 | 4.62E-09 |
| sim | mfcc_sma[2] | 0.02 | 8.45E-03 | 6.39E-03 |
| sim | mfcc_sma[3] | 0.04 | 9.02E-03 | 9.08E-07 |
| sim | mfcc_sma[4] | 0.04 | 8.62E-03 | 2.61E-05 |
| sim | mfcc_sma[5] | -0.04 | 8.66E-03 | 2.60E-06 |
| sim | mfcc_sma[6] | 0.05 | 8.59E-03 | 3.24E-08 |
| sim | mfcc_sma[7] | -0.04 | 8.27E-03 | 3.74E-06 |
| sim | mfcc_sma[8] | 0.05 | 8.65E-03 | 4.58E-08 |
| sim | mfcc_sma[9] | 0.00 | 8.95E-03 | 9.26E-01 |
| sim | mfcc_sma[10] | -0.04 | 8.33E-03 | 8.73E-07 |
| sim | mfcc_sma[11] | 0.05 | 8.39E-03 | 2.38E-10 |
| sim | mfcc_sma[12] | -0.03 | 8.44E-03 | 1.68E-04 |
| sim | mfcc_sma[13] | 0.02 | 8.81E-03 | 6.63E-02 |
| sim | mfcc_sma[14] | 0.03 | 8.86E-03 | 7.93E-04 |
|  |  |  |  |  |
| **BNT30** | F0final_sma | 0.00 | 8.09E-03 | 5.75E-01 |
| BNT30 | voicingFinalUnclipped_sma | 0.00 | 6.92E-03 | 9.83E-01 |
| BNT30 | jitterLocal_sma | 0.00 | 7.31E-03 | 6.46E-01 |
| BNT30 | jitterDDP_sma | 0.00 | 7.21E-03 | 8.74E-01 |
| BNT30 | shimmerLocal_sma | 0.00 | 7.58E-03 | 5.87E-01 |
| BNT30 | logHNR_sma | 0.00 | 7.68E-03 | 6.52E-01 |
| BNT30 | audspec_lengthL1norm_sma | -0.03 | 7.32E-03 | 2.55E-04 |
| BNT30 | audspecRasta_lengthL1norm_sma | 0.02 | 8.01E-03 | 4.06E-03 |
| BNT30 | pcm_RMSenergy_sma | -0.01 | 7.39E-03 | 1.12E-01 |
| BNT30 | pcm_zcr_sma | -0.04 | 6.57E-03 | 2.20E-09 |
| BNT30 | audSpec_Rfilt_sma[0] | -0.02 | 7.73E-03 | 4.46E-02 |
| BNT30 | audSpec_Rfilt_sma[1] | -0.01 | 7.83E-03 | 7.10E-02 |
| BNT30 | audSpec_Rfilt_sma[2] | 0.00 | 7.96E-03 | 8.17E-01 |
| BNT30 | audSpec_Rfilt_sma[3] | 0.01 | 7.97E-03 | 3.36E-01 |
| BNT30 | audSpec_Rfilt_sma[4] | 0.02 | 7.84E-03 | 3.96E-02 |
| BNT30 | audSpec_Rfilt_sma[5] | 0.02 | 7.70E-03 | 5.65E-03 |
| BNT30 | audSpec_Rfilt_sma[6] | 0.03 | 7.63E-03 | 3.13E-04 |
| BNT30 | audSpec_Rfilt_sma[7] | 0.03 | 7.53E-03 | 9.71E-05 |
| BNT30 | audSpec_Rfilt_sma[8] | 0.03 | 7.41E-03 | 3.70E-05 |
| BNT30 | audSpec_Rfilt_sma[9] | 0.03 | 7.41E-03 | 2.21E-05 |
| BNT30 | audSpec_Rfilt_sma[10] | 0.03 | 7.42E-03 | 3.95E-05 |
| BNT30 | audSpec_Rfilt_sma[11] | 0.03 | 7.55E-03 | 2.09E-04 |
| BNT30 | audSpec_Rfilt_sma[12] | 0.03 | 7.70E-03 | 6.66E-04 |
| BNT30 | audSpec_Rfilt_sma[13] | 0.02 | 7.89E-03 | 6.68E-03 |
| BNT30 | audSpec_Rfilt_sma[14] | 0.02 | 7.90E-03 | 2.58E-02 |
| BNT30 | audSpec_Rfilt_sma[15] | 0.02 | 7.88E-03 | 2.80E-02 |
| BNT30 | audSpec_Rfilt_sma[16] | 0.01 | 7.90E-03 | 1.25E-01 |
| BNT30 | audSpec_Rfilt_sma[17] | 0.01 | 7.87E-03 | 1.49E-01 |
| BNT30 | audSpec_Rfilt_sma[18] | 0.01 | 7.79E-03 | 2.45E-01 |
| BNT30 | audSpec_Rfilt_sma[19] | 0.01 | 7.64E-03 | 1.12E-01 |
| BNT30 | audSpec_Rfilt_sma[20] | 0.01 | 7.69E-03 | 6.18E-02 |
| BNT30 | audSpec_Rfilt_sma[21] | 0.02 | 7.86E-03 | 3.99E-02 |
| BNT30 | audSpec_Rfilt_sma[22] | 0.01 | 7.61E-03 | 1.09E-01 |
| BNT30 | audSpec_Rfilt_sma[23] | 0.00 | 7.37E-03 | 7.00E-01 |
| BNT30 | audSpec_Rfilt_sma[24] | 0.00 | 7.26E-03 | 6.23E-01 |
| BNT30 | audSpec_Rfilt_sma[25] | 0.00 | 7.29E-03 | 9.05E-01 |
| BNT30 | pcm_fftMag_fband250-650_sma | 0.01 | 7.26E-03 | 1.88E-01 |
| BNT30 | pcm_fftMag_fband1000-4000_sma | -0.02 | 7.50E-03 | 1.54E-02 |
| BNT30 | pcm_fftMag_spectralRollOff25.0_sma | -0.04 | 6.70E-03 | 3.08E-09 |
| BNT30 | pcm_fftMag_spectralRollOff50.0_sma | -0.04 | 6.64E-03 | 1.67E-09 |
| BNT30 | pcm_fftMag_spectralRollOff75.0_sma | -0.04 | 6.63E-03 | 1.68E-09 |
| BNT30 | pcm_fftMag_spectralRollOff90.0_sma | -0.04 | 6.64E-03 | 1.91E-09 |
| BNT30 | pcm_fftMag_spectralFlux_sma | -0.01 | 7.43E-03 | 5.58E-02 |
| BNT30 | pcm_fftMag_spectralCentroid_sma | -0.04 | 6.61E-03 | 1.78E-09 |
| BNT30 | pcm_fftMag_spectralEntropy_sma | -0.04 | 6.63E-03 | 7.27E-10 |
| BNT30 | pcm_fftMag_spectralVariance_sma | -0.04 | 6.61E-03 | 1.13E-07 |
| BNT30 | pcm_fftMag_spectralSkewness_sma | 0.04 | 6.47E-03 | 1.03E-09 |
| BNT30 | pcm_fftMag_spectralKurtosis_sma | 0.03 | 6.64E-03 | 4.03E-06 |
| BNT30 | pcm_fftMag_spectralSlope_sma | -0.01 | 7.82E-03 | 4.86E-01 |
| BNT30 | pcm_fftMag_psySharpness_sma | -0.04 | 6.64E-03 | 1.38E-09 |
| BNT30 | pcm_fftMag_spectralHarmonicity_sma | 0.00 | 7.31E-03 | 8.79E-01 |
| BNT30 | mfcc_sma[1] | 0.02 | 6.74E-03 | 3.30E-04 |
| BNT30 | mfcc_sma[2] | 0.04 | 6.88E-03 | 3.15E-10 |
| BNT30 | mfcc_sma[3] | 0.01 | 7.46E-03 | 3.36E-01 |
| BNT30 | mfcc_sma[4] | 0.04 | 6.95E-03 | 6.29E-08 |
| BNT30 | mfcc_sma[5] | -0.01 | 7.08E-03 | 4.81E-02 |
| BNT30 | mfcc_sma[6] | 0.02 | 6.90E-03 | 1.53E-03 |
| BNT30 | mfcc_sma[7] | 0.00 | 6.79E-03 | 8.80E-01 |
| BNT30 | mfcc_sma[8] | 0.03 | 7.03E-03 | 2.21E-04 |
| BNT30 | mfcc_sma[9] | 0.00 | 7.51E-03 | 8.83E-01 |
| BNT30 | mfcc_sma[10] | 0.00 | 6.81E-03 | 5.28E-01 |
| BNT30 | mfcc_sma[11] | 0.02 | 6.71E-03 | 1.44E-03 |
| BNT30 | mfcc_sma[12] | -0.01 | 6.99E-03 | 2.05E-01 |
| BNT30 | mfcc_sma[13] | 0.02 | 7.36E-03 | 2.80E-02 |
| BNT30 | mfcc_sma[14] | 0.00 | 7.41E-03 | 5.04E-01 |
|  |  |  |  |  |
| **TrailsA** | F0final_sma | 0.01 | 8.98E-03 | 1.28E-01 |
| TrailsA | voicingFinalUnclipped_sma | 0.01 | 7.92E-03 | 3.18E-01 |
| TrailsA | jitterLocal_sma | 0.00 | 8.26E-03 | 5.98E-01 |
| TrailsA | jitterDDP_sma | 0.01 | 8.12E-03 | 3.08E-01 |
| TrailsA | shimmerLocal_sma | 0.00 | 8.52E-03 | 8.30E-01 |
| TrailsA | logHNR_sma | 0.02 | 8.59E-03 | 4.15E-02 |
| TrailsA | audspec_lengthL1norm_sma | 0.03 | 8.16E-03 | 6.50E-04 |
| TrailsA | audspecRasta_lengthL1norm_sma | -0.01 | 9.06E-03 | 1.33E-01 |
| TrailsA | pcm_RMSenergy_sma | 0.02 | 8.20E-03 | 2.59E-02 |
| TrailsA | pcm_zcr_sma | 0.03 | 7.69E-03 | 2.42E-05 |
| TrailsA | audSpec_Rfilt_sma[0] | 0.01 | 8.66E-03 | 1.37E-01 |
| TrailsA | audSpec_Rfilt_sma[1] | 0.03 | 8.96E-03 | 3.57E-04 |
| TrailsA | audSpec_Rfilt_sma[2] | 0.02 | 9.13E-03 | 2.00E-02 |
| TrailsA | audSpec_Rfilt_sma[3] | 0.02 | 9.11E-03 | 7.57E-02 |
| TrailsA | audSpec_Rfilt_sma[4] | 0.00 | 8.95E-03 | 9.57E-01 |
| TrailsA | audSpec_Rfilt_sma[5] | -0.01 | 8.83E-03 | 2.92E-01 |
| TrailsA | audSpec_Rfilt_sma[6] | -0.01 | 8.77E-03 | 1.57E-01 |
| TrailsA | audSpec_Rfilt_sma[7] | -0.02 | 8.64E-03 | 4.19E-02 |
| TrailsA | audSpec_Rfilt_sma[8] | -0.02 | 8.52E-03 | 1.10E-02 |
| TrailsA | audSpec_Rfilt_sma[9] | -0.02 | 8.53E-03 | 1.10E-02 |
| TrailsA | audSpec_Rfilt_sma[10] | -0.02 | 8.54E-03 | 1.16E-02 |
| TrailsA | audSpec_Rfilt_sma[11] | -0.02 | 8.66E-03 | 1.20E-02 |
| TrailsA | audSpec_Rfilt_sma[12] | -0.02 | 8.78E-03 | 7.39E-03 |
| TrailsA | audSpec_Rfilt_sma[13] | -0.02 | 8.95E-03 | 1.65E-02 |
| TrailsA | audSpec_Rfilt_sma[14] | -0.02 | 8.94E-03 | 2.33E-02 |
| TrailsA | audSpec_Rfilt_sma[15] | -0.02 | 8.89E-03 | 1.59E-02 |
| TrailsA | audSpec_Rfilt_sma[16] | -0.02 | 8.86E-03 | 7.48E-02 |
| TrailsA | audSpec_Rfilt_sma[17] | -0.01 | 8.84E-03 | 1.47E-01 |
| TrailsA | audSpec_Rfilt_sma[18] | 0.00 | 8.77E-03 | 6.31E-01 |
| TrailsA | audSpec_Rfilt_sma[19] | 0.00 | 8.64E-03 | 6.70E-01 |
| TrailsA | audSpec_Rfilt_sma[20] | 0.00 | 8.72E-03 | 9.53E-01 |
| TrailsA | audSpec_Rfilt_sma[21] | 0.00 | 8.90E-03 | 8.32E-01 |
| TrailsA | audSpec_Rfilt_sma[22] | 0.00 | 8.63E-03 | 8.77E-01 |
| TrailsA | audSpec_Rfilt_sma[23] | 0.00 | 8.34E-03 | 7.91E-01 |
| TrailsA | audSpec_Rfilt_sma[24] | 0.00 | 8.16E-03 | 6.71E-01 |
| TrailsA | audSpec_Rfilt_sma[25] | 0.00 | 8.18E-03 | 8.13E-01 |
| TrailsA | pcm_fftMag_fband250-650_sma | 0.00 | 8.20E-03 | 6.34E-01 |
| TrailsA | pcm_fftMag_fband1000-4000_sma | 0.03 | 8.27E-03 | 1.11E-04 |
| TrailsA | pcm_fftMag_spectralRollOff25.0_sma | 0.03 | 7.78E-03 | 4.85E-04 |
| TrailsA | pcm_fftMag_spectralRollOff50.0_sma | 0.03 | 7.75E-03 | 2.10E-04 |
| TrailsA | pcm_fftMag_spectralRollOff75.0_sma | 0.03 | 7.74E-03 | 7.55E-05 |
| TrailsA | pcm_fftMag_spectralRollOff90.0_sma | 0.03 | 7.76E-03 | 4.98E-05 |
| TrailsA | pcm_fftMag_spectralFlux_sma | 0.02 | 8.23E-03 | 1.71E-02 |
| TrailsA | pcm_fftMag_spectralCentroid_sma | 0.03 | 7.73E-03 | 6.68E-05 |
| TrailsA | pcm_fftMag_spectralEntropy_sma | 0.03 | 7.76E-03 | 6.01E-05 |
| TrailsA | pcm_fftMag_spectralVariance_sma | 0.03 | 7.73E-03 | 2.28E-05 |
| TrailsA | pcm_fftMag_spectralSkewness_sma | -0.04 | 7.54E-03 | 1.44E-06 |
| TrailsA | pcm_fftMag_spectralKurtosis_sma | -0.03 | 7.71E-03 | 4.48E-05 |
| TrailsA | pcm_fftMag_spectralSlope_sma | -0.01 | 8.85E-03 | 1.88E-01 |
| TrailsA | pcm_fftMag_psySharpness_sma | 0.03 | 7.76E-03 | 1.36E-04 |
| TrailsA | pcm_fftMag_spectralHarmonicity_sma | 0.02 | 8.19E-03 | 1.95E-02 |
| TrailsA | mfcc_sma[1] | -0.03 | 7.81E-03 | 5.77E-04 |
| TrailsA | mfcc_sma[2] | -0.02 | 7.78E-03 | 1.50E-02 |
| TrailsA | mfcc_sma[3] | 0.00 | 8.38E-03 | 7.92E-01 |
| TrailsA | mfcc_sma[4] | -0.02 | 7.94E-03 | 6.24E-03 |
| TrailsA | mfcc_sma[5] | 0.02 | 8.01E-03 | 1.36E-02 |
| TrailsA | mfcc_sma[6] | -0.02 | 7.95E-03 | 2.20E-02 |
| TrailsA | mfcc_sma[7] | 0.01 | 7.69E-03 | 3.08E-01 |
| TrailsA | mfcc_sma[8] | -0.02 | 7.97E-03 | 7.52E-03 |
| TrailsA | mfcc_sma[9] | 0.00 | 8.26E-03 | 9.65E-01 |
| TrailsA | mfcc_sma[10] | 0.01 | 7.73E-03 | 3.28E-01 |
| TrailsA | mfcc_sma[11] | -0.02 | 7.79E-03 | 1.85E-02 |
| TrailsA | mfcc_sma[12] | 0.01 | 7.79E-03 | 8.34E-02 |
| TrailsA | mfcc_sma[13] | -0.01 | 8.11E-03 | 2.08E-01 |
| TrailsA | mfcc_sma[14] | 0.00 | 8.25E-03 | 6.52E-01 |
|  |  |  |  |  |
| **TrailsB** | F0final_sma | 0.01 | 8.92E-03 | 1.61E-01 |
| TrailsB | voicingFinalUnclipped_sma | 0.01 | 7.80E-03 | 1.79E-01 |
| TrailsB | jitterLocal_sma | 0.00 | 8.14E-03 | 8.21E-01 |
| TrailsB | jitterDDP_sma | 0.00 | 7.98E-03 | 6.88E-01 |
| TrailsB | shimmerLocal_sma | 0.00 | 8.42E-03 | 9.82E-01 |
| TrailsB | logHNR_sma | 0.01 | 8.50E-03 | 3.20E-01 |
| TrailsB | audspec_lengthL1norm_sma | 0.02 | 8.06E-03 | 4.10E-02 |
| TrailsB | audspecRasta_lengthL1norm_sma | -0.01 | 8.98E-03 | 1.44E-01 |
| TrailsB | pcm_RMSenergy_sma | 0.01 | 8.10E-03 | 1.42E-01 |
| TrailsB | pcm_zcr_sma | 0.02 | 7.61E-03 | 3.39E-03 |
| TrailsB | audSpec_Rfilt_sma[0] | 0.02 | 8.56E-03 | 4.61E-02 |
| TrailsB | audSpec_Rfilt_sma[1] | 0.03 | 8.91E-03 | 4.28E-03 |
| TrailsB | audSpec_Rfilt_sma[2] | 0.02 | 9.09E-03 | 6.56E-02 |
| TrailsB | audSpec_Rfilt_sma[3] | 0.01 | 9.04E-03 | 3.60E-01 |
| TrailsB | audSpec_Rfilt_sma[4] | 0.00 | 8.87E-03 | 8.75E-01 |
| TrailsB | audSpec_Rfilt_sma[5] | -0.01 | 8.75E-03 | 4.77E-01 |
| TrailsB | audSpec_Rfilt_sma[6] | -0.01 | 8.70E-03 | 2.48E-01 |
| TrailsB | audSpec_Rfilt_sma[7] | -0.01 | 8.58E-03 | 1.15E-01 |
| TrailsB | audSpec_Rfilt_sma[8] | -0.02 | 8.44E-03 | 5.23E-02 |
| TrailsB | audSpec_Rfilt_sma[9] | -0.02 | 8.46E-03 | 4.57E-02 |
| TrailsB | audSpec_Rfilt_sma[10] | -0.01 | 8.46E-03 | 9.38E-02 |
| TrailsB | audSpec_Rfilt_sma[11] | -0.01 | 8.58E-03 | 1.96E-01 |
| TrailsB | audSpec_Rfilt_sma[12] | -0.01 | 8.70E-03 | 1.59E-01 |
| TrailsB | audSpec_Rfilt_sma[13] | -0.01 | 8.87E-03 | 1.16E-01 |
| TrailsB | audSpec_Rfilt_sma[14] | -0.02 | 8.86E-03 | 7.47E-02 |
| TrailsB | audSpec_Rfilt_sma[15] | -0.02 | 8.80E-03 | 3.19E-02 |
| TrailsB | audSpec_Rfilt_sma[16] | -0.02 | 8.77E-03 | 6.35E-02 |
| TrailsB | audSpec_Rfilt_sma[17] | -0.01 | 8.76E-03 | 1.48E-01 |
| TrailsB | audSpec_Rfilt_sma[18] | -0.01 | 8.71E-03 | 3.98E-01 |
| TrailsB | audSpec_Rfilt_sma[19] | -0.01 | 8.58E-03 | 5.01E-01 |
| TrailsB | audSpec_Rfilt_sma[20] | -0.01 | 8.66E-03 | 4.25E-01 |
| TrailsB | audSpec_Rfilt_sma[21] | -0.01 | 8.83E-03 | 3.44E-01 |
| TrailsB | audSpec_Rfilt_sma[22] | -0.01 | 8.55E-03 | 2.99E-01 |
| TrailsB | audSpec_Rfilt_sma[23] | -0.01 | 8.26E-03 | 4.55E-01 |
| TrailsB | audSpec_Rfilt_sma[24] | 0.00 | 8.07E-03 | 5.95E-01 |
| TrailsB | audSpec_Rfilt_sma[25] | 0.00 | 8.12E-03 | 6.41E-01 |
| TrailsB | pcm_fftMag_fband250-650_sma | 0.01 | 8.11E-03 | 2.74E-01 |
| TrailsB | pcm_fftMag_fband1000-4000_sma | 0.02 | 8.20E-03 | 3.38E-02 |
| TrailsB | pcm_fftMag_spectralRollOff25.0_sma | 0.02 | 7.70E-03 | 6.44E-03 |
| TrailsB | pcm_fftMag_spectralRollOff50.0_sma | 0.02 | 7.67E-03 | 4.48E-03 |
| TrailsB | pcm_fftMag_spectralRollOff75.0_sma | 0.02 | 7.66E-03 | 3.47E-03 |
| TrailsB | pcm_fftMag_spectralRollOff90.0_sma | 0.02 | 7.68E-03 | 3.96E-03 |
| TrailsB | pcm_fftMag_spectralFlux_sma | 0.01 | 8.13E-03 | 1.41E-01 |
| TrailsB | pcm_fftMag_spectralCentroid_sma | 0.02 | 7.65E-03 | 3.64E-03 |
| TrailsB | pcm_fftMag_spectralEntropy_sma | 0.02 | 7.67E-03 | 2.22E-03 |
| TrailsB | pcm_fftMag_spectralVariance_sma | 0.02 | 7.64E-03 | 4.80E-03 |
| TrailsB | pcm_fftMag_spectralSkewness_sma | -0.03 | 7.44E-03 | 3.08E-04 |
| TrailsB | pcm_fftMag_spectralKurtosis_sma | -0.02 | 7.67E-03 | 2.78E-03 |
| TrailsB | pcm_fftMag_spectralSlope_sma | -0.01 | 8.76E-03 | 1.88E-01 |
| TrailsB | pcm_fftMag_psySharpness_sma | 0.02 | 7.67E-03 | 3.65E-03 |
| TrailsB | pcm_fftMag_spectralHarmonicity_sma | 0.02 | 8.10E-03 | 5.87E-02 |
| TrailsB | mfcc_sma[1] | -0.01 | 7.73E-03 | 9.24E-02 |
| TrailsB | mfcc_sma[2] | -0.02 | 7.68E-03 | 1.25E-02 |
| TrailsB | mfcc_sma[3] | 0.00 | 8.27E-03 | 9.60E-01 |
| TrailsB | mfcc_sma[4] | -0.02 | 7.84E-03 | 2.68E-02 |
| TrailsB | mfcc_sma[5] | 0.01 | 7.95E-03 | 4.72E-01 |
| TrailsB | mfcc_sma[6] | -0.01 | 7.86E-03 | 1.50E-01 |
| TrailsB | mfcc_sma[7] | -0.01 | 7.61E-03 | 2.18E-01 |
| TrailsB | mfcc_sma[8] | -0.01 | 7.88E-03 | 2.27E-01 |
| TrailsB | mfcc_sma[9] | 0.00 | 8.20E-03 | 7.88E-01 |
| TrailsB | mfcc_sma[10] | 0.00 | 7.64E-03 | 5.54E-01 |
| TrailsB | mfcc_sma[11] | -0.01 | 7.72E-03 | 1.80E-01 |
| TrailsB | mfcc_sma[12] | -0.01 | 7.74E-03 | 5.06E-01 |
| TrailsB | mfcc_sma[13] | -0.01 | 8.04E-03 | 2.24E-01 |
| TrailsB | mfcc_sma[14] | 0.00 | 8.16E-03 | 8.86E-01 |
|  |  |  |  |  |
| **HVOT** | F0final_sma | -0.05 | 9.27E-03 | 3.59E-07 |
| HVOT | voicingFinalUnclipped_sma | -0.02 | 8.03E-03 | 2.52E-02 |
| HVOT | jitterLocal_sma | -0.02 | 8.41E-03 | 4.44E-03 |
| HVOT | jitterDDP_sma | -0.02 | 8.25E-03 | 8.57E-03 |
| HVOT | shimmerLocal_sma | -0.03 | 8.70E-03 | 3.26E-04 |
| HVOT | logHNR_sma | -0.04 | 8.77E-03 | 5.78E-06 |
| HVOT | audspec_lengthL1norm_sma | -0.03 | 8.32E-03 | 2.13E-03 |
| HVOT | audspecRasta_lengthL1norm_sma | 0.00 | 9.29E-03 | 8.99E-01 |
| HVOT | pcm_RMSenergy_sma | -0.03 | 8.37E-03 | 1.46E-03 |
| HVOT | pcm_zcr_sma | -0.02 | 7.80E-03 | 2.97E-02 |
| HVOT | audSpec_Rfilt_sma[0] | -0.02 | 8.88E-03 | 1.49E-02 |
| HVOT | audSpec_Rfilt_sma[1] | -0.03 | 9.20E-03 | 1.26E-03 |
| HVOT | audSpec_Rfilt_sma[2] | -0.02 | 9.35E-03 | 2.24E-02 |
| HVOT | audSpec_Rfilt_sma[3] | -0.02 | 9.31E-03 | 3.52E-02 |
| HVOT | audSpec_Rfilt_sma[4] | -0.01 | 9.14E-03 | 1.51E-01 |
| HVOT | audSpec_Rfilt_sma[5] | -0.01 | 9.00E-03 | 2.69E-01 |
| HVOT | audSpec_Rfilt_sma[6] | 0.00 | 8.94E-03 | 6.16E-01 |
| HVOT | audSpec_Rfilt_sma[7] | 0.00 | 8.82E-03 | 9.51E-01 |
| HVOT | audSpec_Rfilt_sma[8] | 0.00 | 8.69E-03 | 7.00E-01 |
| HVOT | audSpec_Rfilt_sma[9] | 0.00 | 8.70E-03 | 6.88E-01 |
| HVOT | audSpec_Rfilt_sma[10] | 0.00 | 8.71E-03 | 7.68E-01 |
| HVOT | audSpec_Rfilt_sma[11] | 0.00 | 8.83E-03 | 7.31E-01 |
| HVOT | audSpec_Rfilt_sma[12] | 0.01 | 8.97E-03 | 5.17E-01 |
| HVOT | audSpec_Rfilt_sma[13] | 0.00 | 9.17E-03 | 6.73E-01 |
| HVOT | audSpec_Rfilt_sma[14] | 0.00 | 9.16E-03 | 8.30E-01 |
| HVOT | audSpec_Rfilt_sma[15] | 0.01 | 9.11E-03 | 3.99E-01 |
| HVOT | audSpec_Rfilt_sma[16] | 0.01 | 9.09E-03 | 3.06E-01 |
| HVOT | audSpec_Rfilt_sma[17] | 0.01 | 9.07E-03 | 3.49E-01 |
| HVOT | audSpec_Rfilt_sma[18] | 0.01 | 8.99E-03 | 2.95E-01 |
| HVOT | audSpec_Rfilt_sma[19] | 0.01 | 8.86E-03 | 3.07E-01 |
| HVOT | audSpec_Rfilt_sma[20] | 0.00 | 8.94E-03 | 6.20E-01 |
| HVOT | audSpec_Rfilt_sma[21] | 0.00 | 9.13E-03 | 5.92E-01 |
| HVOT | audSpec_Rfilt_sma[22] | -0.01 | 8.85E-03 | 3.19E-01 |
| HVOT | audSpec_Rfilt_sma[23] | -0.01 | 8.54E-03 | 1.37E-01 |
| HVOT | audSpec_Rfilt_sma[24] | -0.02 | 8.36E-03 | 2.77E-02 |
| HVOT | audSpec_Rfilt_sma[25] | -0.02 | 8.40E-03 | 1.19E-02 |
| HVOT | pcm_fftMag_fband250-650_sma | -0.02 | 8.34E-03 | 1.18E-02 |
| HVOT | pcm_fftMag_fband1000-4000_sma | -0.02 | 8.47E-03 | 3.84E-03 |
| HVOT | pcm_fftMag_spectralRollOff25.0_sma | -0.02 | 7.89E-03 | 6.58E-03 |
| HVOT | pcm_fftMag_spectralRollOff50.0_sma | -0.02 | 7.86E-03 | 1.25E-02 |
| HVOT | pcm_fftMag_spectralRollOff75.0_sma | -0.02 | 7.85E-03 | 1.65E-02 |
| HVOT | pcm_fftMag_spectralRollOff90.0_sma | -0.02 | 7.87E-03 | 1.77E-02 |
| HVOT | pcm_fftMag_spectralFlux_sma | -0.02 | 8.40E-03 | 4.11E-03 |
| HVOT | pcm_fftMag_spectralCentroid_sma | -0.02 | 7.84E-03 | 1.75E-02 |
| HVOT | pcm_fftMag_spectralEntropy_sma | -0.02 | 7.86E-03 | 3.62E-02 |
| HVOT | pcm_fftMag_spectralVariance_sma | -0.01 | 7.84E-03 | 1.18E-01 |
| HVOT | pcm_fftMag_spectralSkewness_sma | 0.02 | 7.63E-03 | 5.20E-03 |
| HVOT | pcm_fftMag_spectralKurtosis_sma | 0.01 | 7.87E-03 | 1.14E-01 |
| HVOT | pcm_fftMag_spectralSlope_sma | 0.03 | 9.00E-03 | 3.91E-03 |
| HVOT | pcm_fftMag_psySharpness_sma | -0.02 | 7.86E-03 | 1.51E-02 |
| HVOT | pcm_fftMag_spectralHarmonicity_sma | -0.02 | 8.33E-03 | 2.85E-03 |
| HVOT | mfcc_sma[1] | 0.00 | 7.94E-03 | 9.34E-01 |
| HVOT | mfcc_sma[2] | 0.04 | 7.91E-03 | 2.41E-06 |
| HVOT | mfcc_sma[3] | -0.02 | 8.54E-03 | 2.45E-02 |
| HVOT | mfcc_sma[4] | 0.03 | 8.08E-03 | 2.01E-04 |
| HVOT | mfcc_sma[5] | 0.00 | 8.21E-03 | 9.01E-01 |
| HVOT | mfcc_sma[6] | 0.00 | 8.09E-03 | 8.05E-01 |
| HVOT | mfcc_sma[7] | 0.02 | 7.82E-03 | 4.44E-03 |
| HVOT | mfcc_sma[8] | 0.00 | 8.14E-03 | 8.29E-01 |
| HVOT | mfcc_sma[9] | 0.00 | 8.52E-03 | 8.03E-01 |
| HVOT | mfcc_sma[10] | 0.01 | 7.88E-03 | 1.00E-01 |
| HVOT | mfcc_sma[11] | 0.00 | 7.93E-03 | 9.02E-01 |
| HVOT | mfcc_sma[12] | 0.01 | 8.00E-03 | 2.52E-01 |
| HVOT | mfcc_sma[13] | 0.01 | 8.37E-03 | 2.32E-01 |
| HVOT | mfcc_sma[14] | -0.01 | 8.46E-03 | 8.19E-02 |
|  |  |  |  |  |
| **fas** | F0final_sma | -0.04 | 8.62E-03 | 2.87E-05 |
| fas | voicingFinalUnclipped_sma | -0.02 | 7.44E-03 | 1.63E-03 |
| fas | jitterLocal_sma | -0.01 | 7.81E-03 | 1.34E-01 |
| fas | jitterDDP_sma | -0.01 | 7.66E-03 | 9.66E-02 |
| fas | shimmerLocal_sma | -0.01 | 8.14E-03 | 2.96E-01 |
| fas | logHNR_sma | -0.01 | 8.24E-03 | 6.92E-02 |
| fas | audspec_lengthL1norm_sma | -0.03 | 7.81E-03 | 1.50E-04 |
| fas | audspecRasta_lengthL1norm_sma | 0.03 | 8.59E-03 | 6.50E-04 |
| fas | pcm_RMSenergy_sma | -0.03 | 7.79E-03 | 1.08E-04 |
| fas | pcm_zcr_sma | -0.02 | 7.16E-03 | 3.46E-03 |
| fas | audSpec_Rfilt_sma[0] | -0.01 | 8.24E-03 | 2.22E-01 |
| fas | audSpec_Rfilt_sma[1] | -0.01 | 8.32E-03 | 5.18E-01 |
| fas | audSpec_Rfilt_sma[2] | 0.01 | 8.44E-03 | 2.16E-01 |
| fas | audSpec_Rfilt_sma[3] | 0.01 | 8.51E-03 | 3.19E-01 |
| fas | audSpec_Rfilt_sma[4] | 0.01 | 8.41E-03 | 4.26E-01 |
| fas | audSpec_Rfilt_sma[5] | 0.00 | 8.30E-03 | 6.86E-01 |
| fas | audSpec_Rfilt_sma[6] | 0.00 | 8.25E-03 | 7.45E-01 |
| fas | audSpec_Rfilt_sma[7] | 0.01 | 8.14E-03 | 3.21E-01 |
| fas | audSpec_Rfilt_sma[8] | 0.01 | 8.02E-03 | 1.48E-01 |
| fas | audSpec_Rfilt_sma[9] | 0.01 | 8.03E-03 | 1.09E-01 |
| fas | audSpec_Rfilt_sma[10] | 0.02 | 8.05E-03 | 5.99E-02 |
| fas | audSpec_Rfilt_sma[11] | 0.02 | 8.17E-03 | 2.50E-02 |
| fas | audSpec_Rfilt_sma[12] | 0.03 | 8.29E-03 | 1.28E-03 |
| fas | audSpec_Rfilt_sma[13] | 0.03 | 8.47E-03 | 1.49E-04 |
| fas | audSpec_Rfilt_sma[14] | 0.03 | 8.46E-03 | 4.64E-05 |
| fas | audSpec_Rfilt_sma[15] | 0.04 | 8.44E-03 | 2.62E-07 |
| fas | audSpec_Rfilt_sma[16] | 0.05 | 8.43E-03 | 1.87E-08 |
| fas | audSpec_Rfilt_sma[17] | 0.04 | 8.41E-03 | 4.94E-07 |
| fas | audSpec_Rfilt_sma[18] | 0.04 | 8.34E-03 | 7.60E-07 |
| fas | audSpec_Rfilt_sma[19] | 0.04 | 8.19E-03 | 2.02E-07 |
| fas | audSpec_Rfilt_sma[20] | 0.04 | 8.27E-03 | 1.04E-05 |
| fas | audSpec_Rfilt_sma[21] | 0.03 | 8.45E-03 | 2.66E-03 |
| fas | audSpec_Rfilt_sma[22] | 0.02 | 8.21E-03 | 6.51E-03 |
| fas | audSpec_Rfilt_sma[23] | 0.01 | 7.96E-03 | 8.73E-02 |
| fas | audSpec_Rfilt_sma[24] | 0.01 | 7.83E-03 | 4.20E-01 |
| fas | audSpec_Rfilt_sma[25] | 0.01 | 7.82E-03 | 1.91E-01 |
| fas | pcm_fftMag_fband250-650_sma | -0.03 | 7.66E-03 | 8.99E-05 |
| fas | pcm_fftMag_fband1000-4000_sma | -0.02 | 7.99E-03 | 3.08E-03 |
| fas | pcm_fftMag_spectralRollOff25.0_sma | -0.03 | 7.27E-03 | 1.52E-04 |
| fas | pcm_fftMag_spectralRollOff50.0_sma | -0.02 | 7.23E-03 | 8.91E-04 |
| fas | pcm_fftMag_spectralRollOff75.0_sma | -0.02 | 7.22E-03 | 3.06E-03 |
| fas | pcm_fftMag_spectralRollOff90.0_sma | -0.02 | 7.24E-03 | 2.32E-03 |
| fas | pcm_fftMag_spectralFlux_sma | -0.03 | 7.85E-03 | 3.58E-04 |
| fas | pcm_fftMag_spectralCentroid_sma | -0.02 | 7.21E-03 | 1.74E-03 |
| fas | pcm_fftMag_spectralEntropy_sma | -0.03 | 7.23E-03 | 1.49E-04 |
| fas | pcm_fftMag_spectralVariance_sma | -0.01 | 7.21E-03 | 7.20E-02 |
| fas | pcm_fftMag_spectralSkewness_sma | 0.04 | 6.99E-03 | 7.11E-08 |
| fas | pcm_fftMag_spectralKurtosis_sma | 0.03 | 7.08E-03 | 2.41E-06 |
| fas | pcm_fftMag_spectralSlope_sma | 0.03 | 7.54E-03 | 7.15E-05 |
| fas | pcm_fftMag_psySharpness_sma | -0.03 | 7.23E-03 | 4.32E-04 |
| fas | pcm_fftMag_spectralHarmonicity_sma | -0.03 | 7.73E-03 | 1.39E-05 |
| fas | mfcc_sma[1] | -0.01 | 7.33E-03 | 4.06E-01 |
| fas | mfcc_sma[2] | 0.05 | 7.35E-03 | 3.63E-13 |
| fas | mfcc_sma[3] | -0.01 | 8.02E-03 | 6.16E-02 |
| fas | mfcc_sma[4] | 0.04 | 7.49E-03 | 1.25E-06 |
| fas | mfcc_sma[5] | 0.01 | 7.64E-03 | 1.61E-01 |
| fas | mfcc_sma[6] | 0.00 | 7.49E-03 | 8.64E-01 |
| fas | mfcc_sma[7] | 0.04 | 7.27E-03 | 1.14E-07 |
| fas | mfcc_sma[8] | 0.01 | 7.54E-03 | 1.09E-01 |
| fas | mfcc_sma[9] | 0.01 | 7.98E-03 | 3.70E-01 |
| fas | mfcc_sma[10] | 0.03 | 7.32E-03 | 3.09E-06 |
| fas | mfcc_sma[11] | 0.00 | 7.29E-03 | 5.20E-01 |
| fas | mfcc_sma[12] | 0.02 | 7.44E-03 | 2.37E-02 |
| fas | mfcc_sma[13] | 0.04 | 7.82E-03 | 1.30E-06 |
| fas | mfcc_sma[14] | -0.02 | 7.92E-03 | 7.29E-03 |
|  |  |  |  |  |
| cnt_animal | F0final_sma | -0.02 | 9.44E-03 | 3.72E-02 |
| cnt_animal | voicingFinalUnclipped_sma | -0.03 | 8.34E-03 | 3.74E-04 |
| cnt_animal | jitterLocal_sma | 0.00 | 8.74E-03 | 7.44E-01 |
| cnt_animal | jitterDDP_sma | -0.01 | 8.60E-03 | 5.10E-01 |
| cnt_animal | shimmerLocal_sma | 0.00 | 9.01E-03 | 9.84E-01 |
| cnt_animal | logHNR_sma | -0.01 | 9.09E-03 | 5.17E-01 |
| cnt_animal | audspec_lengthL1norm_sma | -0.04 | 8.61E-03 | 9.98E-06 |
| cnt_animal | audspecRasta_lengthL1norm_sma | 0.04 | 9.52E-03 | 7.51E-06 |
| cnt_animal | pcm_RMSenergy_sma | -0.04 | 8.53E-03 | 9.37E-06 |
| cnt_animal | pcm_zcr_sma | -0.03 | 8.17E-03 | 1.22E-03 |
| cnt_animal | audSpec_Rfilt_sma[0] | -0.01 | 8.96E-03 | 1.20E-01 |
| cnt_animal | audSpec_Rfilt_sma[1] | 0.00 | 9.14E-03 | 6.07E-01 |
| cnt_animal | audSpec_Rfilt_sma[2] | 0.02 | 9.37E-03 | 1.40E-02 |
| cnt_animal | audSpec_Rfilt_sma[3] | 0.02 | 9.44E-03 | 1.56E-02 |
| cnt_animal | audSpec_Rfilt_sma[4] | 0.02 | 9.36E-03 | 9.10E-03 |
| cnt_animal | audSpec_Rfilt_sma[5] | 0.02 | 9.26E-03 | 3.67E-02 |
| cnt_animal | audSpec_Rfilt_sma[6] | 0.02 | 9.23E-03 | 2.60E-02 |
| cnt_animal | audSpec_Rfilt_sma[7] | 0.02 | 9.14E-03 | 8.59E-03 |
| cnt_animal | audSpec_Rfilt_sma[8] | 0.03 | 9.02E-03 | 2.18E-03 |
| cnt_animal | audSpec_Rfilt_sma[9] | 0.03 | 9.04E-03 | 1.11E-03 |
| cnt_animal | audSpec_Rfilt_sma[10] | 0.03 | 9.05E-03 | 1.04E-03 |
| cnt_animal | audSpec_Rfilt_sma[11] | 0.03 | 9.16E-03 | 1.92E-04 |
| cnt_animal | audSpec_Rfilt_sma[12] | 0.04 | 9.27E-03 | 1.40E-06 |
| cnt_animal | audSpec_Rfilt_sma[13] | 0.05 | 9.42E-03 | 3.86E-07 |
| cnt_animal | audSpec_Rfilt_sma[14] | 0.05 | 9.40E-03 | 1.86E-07 |
| cnt_animal | audSpec_Rfilt_sma[15] | 0.06 | 9.36E-03 | 2.32E-09 |
| cnt_animal | audSpec_Rfilt_sma[16] | 0.06 | 9.31E-03 | 2.64E-10 |
| cnt_animal | audSpec_Rfilt_sma[17] | 0.05 | 9.30E-03 | 8.49E-09 |
| cnt_animal | audSpec_Rfilt_sma[18] | 0.05 | 9.23E-03 | 2.11E-08 |
| cnt_animal | audSpec_Rfilt_sma[19] | 0.05 | 9.08E-03 | 2.96E-09 |
| cnt_animal | audSpec_Rfilt_sma[20] | 0.05 | 9.16E-03 | 2.84E-08 |
| cnt_animal | audSpec_Rfilt_sma[21] | 0.03 | 9.34E-03 | 2.21E-04 |
| cnt_animal | audSpec_Rfilt_sma[22] | 0.02 | 9.08E-03 | 2.18E-02 |
| cnt_animal | audSpec_Rfilt_sma[23] | 0.01 | 8.79E-03 | 4.58E-01 |
| cnt_animal | audSpec_Rfilt_sma[24] | -0.01 | 8.62E-03 | 3.78E-01 |
| cnt_animal | audSpec_Rfilt_sma[25] | 0.00 | 8.60E-03 | 7.97E-01 |
| cnt_animal | pcm_fftMag_fband250-650_sma | -0.03 | 8.46E-03 | 4.07E-04 |
| cnt_animal | pcm_fftMag_fband1000-4000_sma | -0.03 | 8.70E-03 | 2.03E-04 |
| cnt_animal | pcm_fftMag_spectralRollOff25.0_sma | -0.03 | 8.25E-03 | 9.80E-05 |
| cnt_animal | pcm_fftMag_spectralRollOff50.0_sma | -0.03 | 8.22E-03 | 4.08E-04 |
| cnt_animal | pcm_fftMag_spectralRollOff75.0_sma | -0.03 | 8.22E-03 | 8.12E-04 |
| cnt_animal | pcm_fftMag_spectralRollOff90.0_sma | -0.03 | 8.24E-03 | 3.33E-04 |
| cnt_animal | pcm_fftMag_spectralFlux_sma | -0.04 | 8.59E-03 | 1.83E-05 |
| cnt_animal | pcm_fftMag_spectralCentroid_sma | -0.03 | 8.21E-03 | 5.63E-04 |
| cnt_animal | pcm_fftMag_spectralEntropy_sma | -0.04 | 8.25E-03 | 1.84E-05 |
| cnt_animal | pcm_fftMag_spectralVariance_sma | -0.02 | 8.19E-03 | 6.59E-02 |
| cnt_animal | pcm_fftMag_spectralSkewness_sma | 0.04 | 7.99E-03 | 1.99E-08 |
| cnt_animal | pcm_fftMag_spectralKurtosis_sma | 0.04 | 8.07E-03 | 3.21E-06 |
| cnt_animal | pcm_fftMag_spectralSlope_sma | 0.03 | 8.39E-03 | 1.26E-04 |
| cnt_animal | pcm_fftMag_psySharpness_sma | -0.03 | 8.24E-03 | 1.62E-04 |
| cnt_animal | pcm_fftMag_spectralHarmonicity_sma | -0.03 | 8.50E-03 | 4.08E-05 |
| cnt_animal | mfcc_sma[1] | -0.01 | 8.26E-03 | 1.58E-01 |
| cnt_animal | mfcc_sma[2] | 0.07 | 8.19E-03 | 2.59E-18 |
| cnt_animal | mfcc_sma[3] | -0.04 | 8.83E-03 | 1.72E-05 |
| cnt_animal | mfcc_sma[4] | 0.06 | 8.40E-03 | 3.81E-11 |
| cnt_animal | mfcc_sma[5] | 0.00 | 8.49E-03 | 9.59E-01 |
| cnt_animal | mfcc_sma[6] | 0.00 | 8.42E-03 | 7.24E-01 |
| cnt_animal | mfcc_sma[7] | 0.04 | 8.07E-03 | 2.81E-08 |
| cnt_animal | mfcc_sma[8] | 0.01 | 8.44E-03 | 3.35E-01 |
| cnt_animal | mfcc_sma[9] | 0.02 | 8.72E-03 | 2.04E-02 |
| cnt_animal | mfcc_sma[10] | 0.04 | 8.13E-03 | 4.82E-08 |
| cnt_animal | mfcc_sma[11] | 0.00 | 8.22E-03 | 8.15E-01 |
| cnt_animal | mfcc_sma[12] | 0.03 | 8.22E-03 | 1.62E-03 |
| cnt_animal | mfcc_sma[13] | 0.03 | 8.57E-03 | 1.03E-04 |
| cnt_animal | mfcc_sma[14] | -0.02 | 8.68E-03 | 4.45E-03 |

**Table S3**. Cognitive domains and associated acoustic features

| **Cognitive domain** | **NP tests** | **Number of significant features** | **Significant acoustic features** |
| --- | --- | --- | --- |
| Verbal memory | LMi  LMr  LMd  PASi  PASd  PASr | 14 | voicingFinalUnclipped_sma_mean  audSpec_Rfilt_sma[0]_mean  audSpec_Rfilt_sma[1]_mean  audSpec_Rfilt_sma[2]_mean  audSpec_Rfilt_sma[23]_mean  audSpec_Rfilt_sma[24]_mean  audSpec_Rfilt_sma[25]_mean  pcm_fftMag_spectralRollOff50.0_sma_mean  pcm_fftMag_spectralRollOff75.0_sma_mean  mfcc_sma[4]_mean  mfcc_sma[8]_mean  mfcc_sma[14]_mean  pcm_fftMag_spectralCentroid_sma_mean  pcm_fftMag_spectralSkewness_sma_mean |
| Verbal fluency | FAS  CNT_Animal | 35 | voicingFinalUnclipped_sma_mean  audSpec_Rfilt_sma[11]_mean  audSpec_Rfilt_sma[12]_mean  audSpec_Rfilt_sma[13]_mean  audSpec_Rfilt_sma[14]_mean  audSpec_Rfilt_sma[15]_mean  audSpec_Rfilt_sma[16]_mean  audSpec_Rfilt_sma[17]_mean  audSpec_Rfilt_sma[18]_mean  audSpec_Rfilt_sma[19]_mean  audSpec_Rfilt_sma[20]_mean  audSpec_Rfilt_sma[21]_mean  pcm_fftMag_spectralHarmonicity_sma_mean  pcm_fftMag_spectralSlope_sma_mean  pcm_fftMag_spectralSkewness_sma_mean  pcm_fftMag_spectralRollOff50.0_sma_mean  pcm_fftMag_spectralKurtosis_sma_mean  pcm_fftMag_spectralEntropy_sma_mean  pcm_fftMag_spectralFlux_sma_mean  pcm_fftMag_spectralCentroid_sma_mean  pcm_fftMag_fband1000-4000_sma_mean  pcm_fftMag_fband250-650_sma_mean  pcm_fftMag_spectralRollOff25.0_sma_mean  pcm_fftMag_psySharpness_sma_mean  pcm_fftMag_spectralRollOff90.0_sma_mean  pcm_RMSenergy_sma_mean  audspecRasta_lengthL1norm_sma_mean  audspec_lengthL1norm_sma_mean  mfcc_sma[2]_mean  mfcc_sma[3]_mean  mfcc_sma[4]_mean  mfcc_sma[7]_mean  mfcc_sma[10]_mean  mfcc_sma[13]_mean  F0final_sma_mean |
| Visual memory | VRi  VRd  VRr | 49 | voicingFinalUnclipped_sma_mean  audSpec_Rfilt_sma[3]_mean  audSpec_Rfilt_sma[4]_mean  audSpec_Rfilt_sma[5]_mean  audSpec_Rfilt_sma[6]_mean  audSpec_Rfilt_sma[7]_mean  audSpec_Rfilt_sma[8]_mean  audSpec_Rfilt_sma[9]_mean  audSpec_Rfilt_sma[10]_mean  audSpec_Rfilt_sma[11]_mean  audSpec_Rfilt_sma[12]_mean  audSpec_Rfilt_sma[13]_mean  audSpec_Rfilt_sma[14]_mean  audSpec_Rfilt_sma[15]_mean  audSpec_Rfilt_sma[21]_mean  audSpec_Rfilt_sma[23]_mean  audSpec_Rfilt_sma[24]_mean  audSpec_Rfilt_sma[25]_mean  pcm_zcr_sma_mean  shimmerLocal_sma_mean  mfcc_sma[1]_mean  mfcc_sma[2]_mean  mfcc_sma[3]_mean  mfcc_sma[4]_mean  mfcc_sma[5]_mean  mfcc_sma[6]_mean  mfcc_sma[7]_mean  mfcc_sma[8]_mean  mfcc_sma[10]_mean  mfcc_sma[11]_mean  mfcc_sma[12]_mean  mfcc_sma[14]_mean  jitterLocal_sma_mean  jitterDDP_sma_mean  audspecRasta_lengthL1norm_sma_mean  audspec_lengthL1norm_sma_mean  pcm_fftMag_spectralSlope_sma_mean  pcm_fftMag_spectralSkewness_sma_mean  pcm_fftMag_spectralRollOff50.0_sma_mean  pcm_fftMag_spectralKurtosis_sma_mean  pcm_fftMag_spectralEntropy_sma_mean  pcm_fftMag_spectralCentroid_sma_mean  pcm_fftMag_fband1000-4000_sma_mean  pcm_fftMag_fband250-650_sma_mean  pcm_fftMag_spectralRollOff25.0_sma_mean  pcm_fftMag_psySharpness_sma_mean  pcm_fftMag_spectralRollOff90.0_sma_mean  pcm_fftMag_spectralVariance_sma_mean  pcm_fftMag_spectralRollOff75.0_sma_mean |
| Attention and concentration | DSF  Trails A | 48 | shimmerLocal_sma_mean  pcm_fftMag_spectralRollOff75.0_sma_mean  pcm_fftMag_spectralHarmonicity_sma_mean  pcm_fftMag_spectralVariance_sma_mean  pcm_fftMag_spectralSlope_sma_mean  pcm_fftMag_spectralSkewness_sma_mean  pcm_fftMag_spectralRollOff50.0_sma_mean  pcm_fftMag_spectralEntropy_sma_mean  pcm_fftMag_spectralKurtosis_sma_mean  pcm_fftMag_spectralCentroid_sma_mean  pcm_fftMag_fband1000-4000_sma_mean  pcm_fftMag_fband250-650_sma_mean  pcm_fftMag_spectralRollOff25.0_sma_mean  pcm_fftMag_psySharpness_sma_mean  pcm_fftMag_spectralRollOff90.0_sma_mean  pcm_RMSenergy_sma_mean  audSpec_Rfilt_sma[0]_mean  audSpec_Rfilt_sma[1]_mean  audSpec_Rfilt_sma[2]_mean  audSpec_Rfilt_sma[3]_mean  audSpec_Rfilt_sma[4]_mean  audSpec_Rfilt_sma[5]_mean  audSpec_Rfilt_sma[6]_mean  audSpec_Rfilt_sma[7]_mean  audSpec_Rfilt_sma[8]_mean  audSpec_Rfilt_sma[9]_mean  audSpec_Rfilt_sma[10]_mean  audSpec_Rfilt_sma[11]_mean  audSpec_Rfilt_sma[12]_mean  audSpec_Rfilt_sma[13]_mean  audSpec_Rfilt_sma[14]_mean  audSpec_Rfilt_sma[15]_mean  audSpec_Rfilt_sma[21]_mean  audSpec_Rfilt_sma[22]_mean  mfcc_sma[1]_mean  mfcc_sma[3]_mean  mfcc_sma[5]_mean  mfcc_sma[6]_mean  mfcc_sma[7]_mean  mfcc_sma[8]_mean  mfcc_sma[10]_mean  mfcc_sma[11]_mean  mfcc_sma[12]_mean  jitterLocal_sma_mean  audspecRasta_lengthL1norm_sma_mean  audspec_lengthL1norm_sma_mean  F0final_sma_mean  pcm_zcr_sma_mean |
| Executive  function | DSB  Trails B | 30 | audSpec_Rfilt_sma[2]_mean  audSpec_Rfilt_sma[3]_mean  audSpec_Rfilt_sma[4]_mean  audSpec_Rfilt_sma[5]_mean  audSpec_Rfilt_sma[6]_mean  audSpec_Rfilt_sma[7]_mean  audSpec_Rfilt_sma[8]_mean  audSpec_Rfilt_sma[9]_mean  audSpec_Rfilt_sma[10]_mean  audSpec_Rfilt_sma[11]_mean  audSpec_Rfilt_sma[12]_mean  audSpec_Rfilt_sma[13]_mean  audSpec_Rfilt_sma[14]_mean  audSpec_Rfilt_sma[21]_mean  audspecRasta_lengthL1norm_sma_mean  pcm_fftMag_spectralRollOff75.0_sma_mean  pcm_fftMag_spectralSkewness_sma_mean  pcm_fftMag_spectralRollOff50.0_sma_mean  pcm_fftMag_spectralEntropy_sma_mean  pcm_fftMag_spectralCentroid_sma_mean  pcm_fftMag_fband250-650_sma_mean  pcm_fftMag_spectralRollOff25.0_sma_mean  pcm_fftMag_psySharpness_sma_mean  pcm_fftMag_spectralRollOff90.0_sma_mean  pcm_fftMag_spectralVariance_sma_mean  mfcc_sma[1]_mean  mfcc_sma[8]_mean  mfcc_sma[11]_mean  mfcc_sma[12]_mean  pcm_zcr_sma_mean |
| Abstract  reasoning | SIM | 24 | pcm_fftMag_spectralRollOff75.0_sma_mean  pcm_fftMag_spectralVariance_sma_mean  pcm_fftMag_spectralSkewness_sma_mean  pcm_fftMag_spectralRollOff50.0_sma_mean  pcm_fftMag_spectralEntropy_sma_mean  pcm_fftMag_spectralCentroid_sma_mean  pcm_fftMag_spectralRollOff25.0_sma_mean  pcm_fftMag_psySharpness_sma_mean  pcm_fftMag_spectralRollOff90.0_sma_mean  mfcc_sma[1]_mean  mfcc_sma[3]_mean  mfcc_sma[4]_mean  mfcc_sma[5]_mean  mfcc_sma[6]_mean  mfcc_sma[7]_mean  mfcc_sma[8]_mean  mfcc_sma[10]_mean  mfcc_sma[11]_mean  mfcc_sma[12]_mean  audSpec_Rfilt_sma[9]_mean  audSpec_Rfilt_sma[10]_mean  audSpec_Rfilt_sma[24]_mean  audSpec_Rfilt_sma[25]_mean  pcm_zcr_sma_mean |
| Visuoperceptual  organization | HVOT | 5 | mfcc_sma[2]_mean  mfcc_sma[4]_mean  shimmerLocal_sma_mean  logHNR_sma_mean  F0final_sma_mean |
| Language | BNT30 | 23 | audSpec_Rfilt_sma[6]_mean  audSpec_Rfilt_sma[7]_mean  audSpec_Rfilt_sma[8]_mean  audSpec_Rfilt_sma[9]_mean  audSpec_Rfilt_sma[10]_mean  audSpec_Rfilt_sma[11]_mean  audSpec_Rfilt_sma[12]_mean  pcm_fftMag_spectralRollOff75.0_sma_mean  pcm_fftMag_spectralVariance_sma_mean  pcm_fftMag_spectralSkewness_sma_mean  pcm_fftMag_spectralRollOff50.0_sma_mean  pcm_fftMag_spectralKurtosis_sma_mean  pcm_fftMag_spectralEntropy_sma_mean  pcm_fftMag_spectralCentroid_sma_mean  pcm_fftMag_spectralRollOff25.0_sma_mean  pcm_fftMag_psySharpness_sma_mean  pcm_fftMag_spectralRollOff90.0_sma_mean  mfcc_sma[1]_mean  mfcc_sma[2]_mean  mfcc_sma[4]_mean  mfcc_sma[8]_mean  audspec_lengthL1norm_sma_mean  pcm_zcr_sma_mean |

**Table S4**. Number of significant acoustic features with corresponding NP tests

| **NP test** | **Significant acoustic features, *n*** | |
| --- | --- | --- |
|  | **Primary model** | **Sensitivity analysis model** |
| LMi | 7 | 4 |
| LMd | 3 | 3 |
| LMr | 3 | 4 |
| VRi | 49 | 50 |
| VRd | 43 | 44 |
| VRr | 10 | 0 |
| PASi | 0 | 0 |
| PASd | 0 | 1 |
| PASr | 7 | 2 |
| DSf | 44 | 42 |
| DSb | 30 | 23 |
| SIM | 24 | 23 |
| BNT30 | 23 | 0 |
| TrailsA | 15 | 1 |
| TrailsB | 1 | 0 |
| HVOT | 5 | 2 |
| FAS | 26 | 17 |
| CNT_Animal | 34 | 16 |

Primary model was adjusted for age, sex, and education.

Sensitivity analysis model was additionally adjusted for BMI, smoking, and employment status.

**Table S5.** The change of median of correlation coefficients between NP tests and acoustic features in two exams. For each NP test conducted at the first exam, we compared its correlation with acoustic features collected at the first exam and the second exam.

| **NP test** | **Difference in median of correlation coefficient** |
| --- | --- |
| LMi | -4.19E-03 |
| LMd | 6.50E-05 |
| LMr | -8.29E-03 |
| VRi | 3.29E-02 |
| VRd | 1.44E-02 |
| VRr | -8.64E-03 |
| PASi | -3.21E-03 |
| PASd | -3.28E-02 |
| PASr | 1.15E-02 |
| DSf | -1.22E-02 |
| DSb | -3.94E-02 |
| SIM | 2.57E-03 |
| BNT30 | -3.98E-03 |
| TrailsA | 7.16E-02 |
| TrailsB | 6.78E-02 |
| HVOT | -1.67E-02 |
| FAS | 3.47E-02 |
| CNT_Animal | -3.45E-02 |

**Figure S1.** The ROC curves of model2 and model 3 to predict incident MCI.


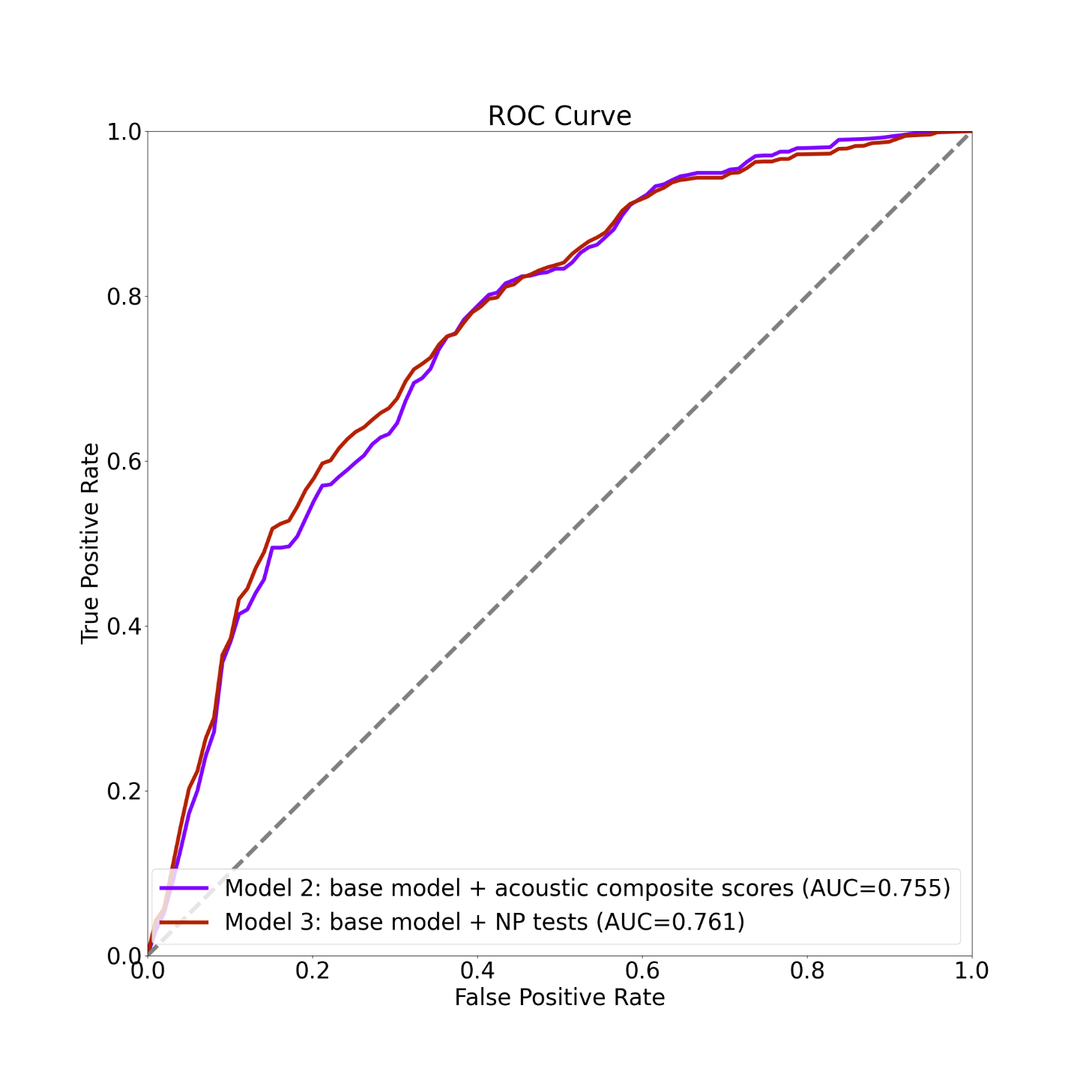

Supplement: Multimedia Appendix 1 [file jmir_v24i12e42886_app1.docx]
